# Genome-Wide Identification, Expansion Mechanism and Expression Profiling Analysis of *GLABROUS1 Enhancer-Binding Protein* *(GeBP)* Gene Family in Gramineae Crops

**DOI:** 10.3390/ijms22168758

**Published:** 2021-08-15

**Authors:** Jishuai Huang, Qiannan Zhang, Yurong He, Wei Liu, Yanghong Xu, Kejia Liu, Fengjun Xian, Junde Li, Jun Hu

**Affiliations:** 1 State Key Laboratory of Hybrid Rice, Engineering Research Center for Plant Biotechnology and Germplasm Utilization of Ministry of Education, College of Life Sciences, Wuhan University, Wuhan 430072, China; huangjishuai28@whu.edu.cn (J.H.); qiannanzhang813@whu.edu.cn (Q.Z.); heyurong@whu.edu.cn (Y.H.); 2017300030057@whu.edu.cn (K.L.); 2019202040064@whu.edu.cn (F.X.); 2016301060020@whu.edu.cn (J.L.); 2 School of Biological Science, University of Bristol, Bristol BS8 1TQ, UK; gz19065@bristol.ac.up; 3 Institute of Neuroscience, State Key Laboratory of Neuroscience, Center for Excellence in Brain Science and Intelligence Technology, Chinese Academy of Sciences, Shanghai 200000, China; xuyh@ion.ac.cn

**Keywords:** Gramineae, *GeBP* genes family, transcription factor, phylogenetic analysis, stress response

## Abstract

The *GLABROUS1 enhancer-binding protein (GeBP)* gene family encodes a typical transcription factor containing a noncanonical Leucine (Leu-)-zipper motif that plays an essential role in regulating plant growth and development, as well as responding to various stresses. However, limited information on the *GeBP* gene family is available in the case of the Gramineae crops. Here, 125 *GeBP* genes from nine Gramineae crops species were phylogenetically classified into four clades using bioinformatics analysis. Evolutionary analyses showed that whole genome duplication (WGD) and segmental duplication play important roles in the expansion of the *GeBP* gene family. The various gene structures and protein motifs revealed that the *GeBP* genes play diverse functions in plants. In addition, the expression profile analysis of the *GeBP* genes showed that 13 genes expressed in all tested organs and stages of development in rice, with especially high levels of expression in the leaf, palea, and lemma. Furthermore, the hormone- and metal-induced expression patterns showed that the expression levels of most genes were affected by various biotic stresses, implying that the *GeBP* genes had an important function in response to various biotic stresses. Furthermore, we confirmed that OsGeBP11 and OsGeBP12 were localized to the nucleus through transient expression in the rice protoplast, indicating that *GeBPs* function as transcription factors to regulate the expression of downstream genes. This study provides a comprehensive understanding of the origin and evolutionary history of the *GeBP* genes family in Gramineae, and will be helpful in a further functional characterization of the *GeBP* genes.

## 1. Introduction 

Transcription factors (TFs) are key regulators in plants, which play crucial roles in various growth and developmental processes, as well as in response to abiotic stresses [1,2,3,4]. Previous studies have systematically identified 129,288 TFs from 83 species, and more than 2000 TFs were identified in rice [5]. However, merely 60 TF families in plants had been reported and identified [6]. Thus, knowledge about some important yet unknown transcription factor families remains elusive. The *GLABROUS1 enhancer-binding protein (GeBP)* gene, first identified and isolated from *Arabidopsis* in 2003, was one of the new plant-specific transcription factor families whose members share a central DNA-binding domain [7]. Although some members of the GeBP gene family have been identified in *Arabidopsis*, their biological functions have not been characterized. 

The trichomes in the epidermis are hair-like structures and constitute the aerial part of most terrestrial plants [8]. In *Arabidopsis*, its initiation requires the activity of the GLABROUS1 (GL1), which is expressed in the epidermis [9]. Curaba et al. have identified and isolated the GL1 enhancer-binding protein (GeBP), which specifically binds to the regulatory element of the promoter and regulates the expression of GL1 [7]. GeBP and its homologs in *Arabidopsis* share two conserved regions, a central region with unknown motifs and a C-terminal region with a putative leucine-zipper motif. Both the regions are necessary for the trans-activation of the downstream gene expression. GeBP is predicted to play a role in various hormonal pathways [10]. The GL1 contains an MYB domain which can be upregulated by the gibberellin (GA) hormones. In the GA-deficient mutant *ga1-3*, the GL1 transcript level significantly decreases, and the rosette leaves are glabrous. The application of exogenous GAs can restore the GL1 expression and rescue the development of trichomes, suggesting that GeBP is also regulated by GAs [11]. A previous report suggested that GA and cytokinin (CK) act antagonistically in leaf formation and meristem maintenance; GA counteracts some effects of the cytokinin during epidermal differentiation [12,13]. However, both of them can stimulate the initiation of trichomes in *Arabidopsis*. In the cytokinin pathway, the production of trichomes requires two genes expressed in the late inflorescence organs, ZFP8 and GIS2, which encode C2H2 transcription factors related to the expression level of GLABROUS INFLORESCENCE STEMS (GIS) [14,15]. GIS2 acts upstream of GL1 and positively regulates its expression on the transcriptional level. Meanwhile, GIS2 can also be induced by cytokinin, which suggests that it also plays an essential role in cytokinin response. Furthermore, the transcript levels of *GeBP* are positively regulated by BREVIPEDICELLUS, a gene of the KNOX family regulating the cytokinin pathway positively in the shoot apical meristem [16,17]. Additionally, in 2008, Gilles Vachon et al. also reported that GeBP/GPL played a redundant role in the cytokinin hormone pathway. It is reported that a triple loss-of-function mutant of *gebp gpl1 gpl2* showed reduced sensitivity to exogenous cytokinin in a subset of cytokinin responses, such as senescence and growth. Meanwhile, the transcription levels of type-A ARR cytokinin response genes were significantly increased in the triple mutant, indicating that GeBP potentially causes an increasingly negative feedback regulation, and ultimately cytokinin insensitivity [18]. In general, GAs and auxin act antagonistically to cytokinin; GAs promote cell differentiation and auxin promotes organ initiation [13,19,20]. However, it is still unclear whether auxin is involved in the formation of trichomes and affects the expression of GeBP family genes.

The root system of plants is particularly plastic during development, and this allows plants to adapt to complex environmental changes [21]. When plants encounter sub-lethal levels of abiotic stresses, such as heavy metal pollutants, the roots exhibit avoidance by changing the root architecture [22]. The GeBP-LIKE 4 (GPL4) transcription factor, identified as an inhibitor of root growth is induced rapidly in root tips in the response to cadmium (Cd) in *Arabidopsis thaliana*. Deepa Khare et al. reported that GPL4 inhibits the growth of roots by modulating the reactive oxygen species (ROS) concentrations when the plants are exposed to toxic metals, thereby facilitating the roots to colonize in the non-contaminated regions of the rhizosphere [21]. Moreover, the root avoidance of GPL4 is not only limited to the response to Cd stress, but also required for response to excess levels of copper (Cu) and zinc (Zn) in *Arabidopsis*. 

The *CONSTITUTIVE EXPRESSOR OF PATHOGENESIS-RELATED GENES5 (CPR5)* gene characterized in *Arabidopsis* encodes a putative membrane protein and has pleiotropic functions, particularly in pathogen responses, cell proliferation, cell expansion, and cell death [23,24,25]. Gilles Vachon et al. reported a link between CPR5 and the GeBP family in 2011. GeBP/GPLs regulated a set of genes in the CPR5 pathway [26]. By analyzing the transcriptomic profile of the *gebp gpl 1, 2, 3* quadruple mutant and the overexpressing line of GPL2, researchers have characterized that the *GeBP/GPLs* genes are involved in defense responses and cell wall metabolism. These genes partially overlap with a subset of CPR5-regulated genes. The transcript levels of the pathogen response marker genes PR1 and PR5 are increased in the mutant, indicating that the GeBP/GPLs are repressors of PR genes. A recent study revealed that VirF and its plant functional homolog VBF of the *Agrobacterium* F-box effector interact with the *Arabidopsis* GeBP-like transcription factor VFP4 [27]. Loss-of-function mutation of VFP4 results in the differential expression of numerous biotic stress response genes, suggesting that one of the functions of VFP4 is to control a spectrum of plant defenses, including against *Agrobacterium tumefaciens*. 

The Gramineae crops, such as rice, maize, and wheat, have high economic values and abundant nutritional values [28]. They are widely used in scientific research because of the availability of abundant diverse genetic resources and high-quality genome reference sequencing data [29]. Recently, many GeBP genes have been characterized in *Arabidopsis*. However, little is known about the evolutionary dynamics of the GeBP family in Gramineae crops. In this study, we have systematically and comprehensively characterized the GeBP gene family in nine Gramineae crops (*Brachypodium distachyon, Hordeum vulgare, Oryza. sativa* ssp. *Indica, Oryza. sativa* ssp. *japonica, Oryza rufipogon, Sorghum bicolor, Setaria* *italica, Triticum aestivum, Zea mays*) using bioinformatics analysis. We have analyzed the chromosomal distributions, phylogenetic relationships, duplication events, orthologous groups, selective forces, gene structure, and protein motifs of all the 125 GeBP genes. Additionally, expression analysis was performed in rice to characterize the functional differentiation of the GeBP gene family. Our study, therefore, lays a foundation for further functional characterization of the GeBP gene family in Gramineae crops. 

## 2. Results 

### 2.1. Identification, Phylogenetic Analysis, and Classification in the Gramineae Crops

**Figure 1:**
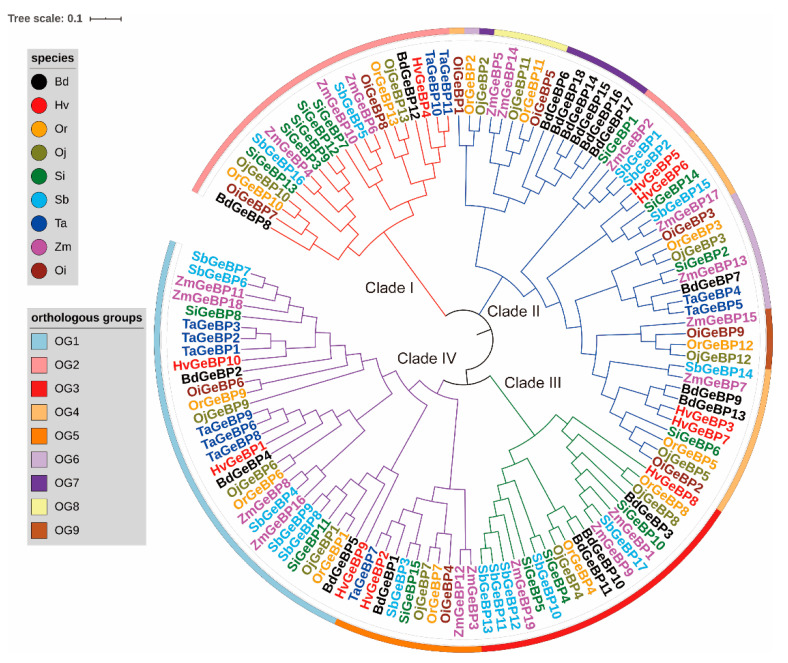
A neighbor-joining (NJ) phylogeny tree of the GeBP protein sequences from *O. rufipogon, O. sativa* ssp. *japonica, Z. mays, H. vulgare, B.distachyon, O. sativa* ssp. *indica, S. bicolor, S. italic,* and *T. aestivum*. The different colors of the branches represent different clades. The colors of the outside circles represent different orthologous groups (OGs), such as OG1. The different species are also displayed by different color markers, such as black denoting Bd.

A total of 125 *GeBP* genes, including 13, 13, 19, 10, 18, 9, 17, 15, and 11 *GeBP* genes, were identified in *O. rufipogon* (Or), *O. sativa* ssp. *japonica* (Oj), *Z. mays* (Zm), *H. vulgare* (Hv), *B. distachyon* (Bd), *O. sativa* ssp. *indica* (Oi), *S. bicolor* (Sb), *S. italica* (Si), and *T. aestivum* (Ta), respectively (Figure S1 and Table S2). We found that Bd, Sb, Si, and Zm had more *GeBP* gene members compared to Hv, Oi, Oj, Or, and Ta, implying a gene expansion of the *GeBP* family among different species. Furthermore, the phylogenetic tree was constructed based on the alignment of the GeBP protein sequences with the neighbor-joining (NJ) method. Results showed that the GeBP proteins were classified into four clades, including Clade I, Clade II, Clade III, and Clade IV (Figure 1). All clades showed a clear expansion of gene numbers, while Clade II and Clade IV had more genes than Clade I and Clade III, and Clade III was absent in Oi and Ta. In addition, we calculated the values of Tajima’D for each clade and found that the Tajima’D values ranged from the lowest, −0.65172 (Clade II) to the highest, 0.18654 (Clade I) (Table S2). Those results indicated that two directional selective sweeps were observed for *GeBP* genes in nine crops. Taken together, these results suggested that the *GeBP* gene family showed different expansion mechanisms in the Gramineae crops during evolution.

To investigate the evolutionary pattern of GeBP genes in these species, the orthologous groups (OGs) were identified by OrthoFinder software 9 (Table S3). The results showed that 125 genes were divided into 9 OGs, and the gene number of each OGs was greatly varying. For example, the OG1, OG2, and OG3 contained 30, 25, and 19 genes, while the OG7, OG8, and OG9 included 7, 5, and 4 genes. In addition, among all the OGs, only three OGs (OG1, OG1, and OG5) were presented in all the Gramineae species, while other OGs were dispersed in the individual species. Interestingly, the 13 genes of Oj contained all OGs. These results suggested that unequal loss and expansion of OGs appeared during the evolutionary process. To characterize the selective pressure on the *GeBP* genes during the evolutionary process, the Tajima’s D was calculated for the orthologous gene pairs among the tested Gramineae crops. We found most of the OG pairs with Tajima’s D < 0, indicating that the *GeBP* gene family might have undergone purifying selection in the evolutionary process.

### 2.2. The Expansion and Evolutionary Pattern of the GeBP Genes 

**Figure 2:**
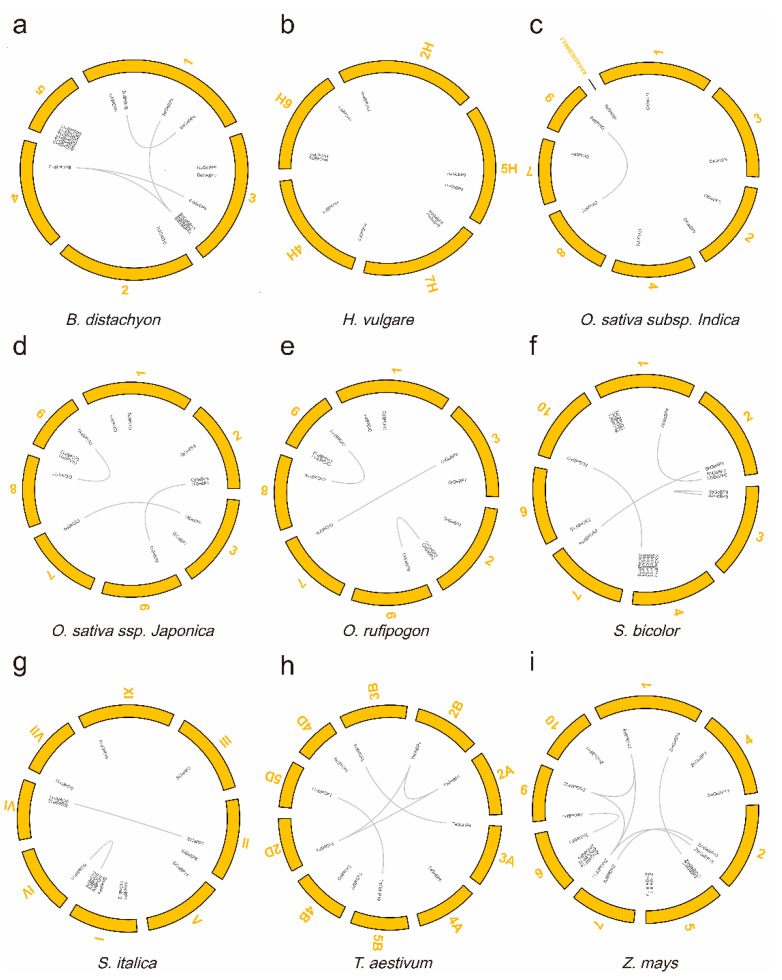
The chromosomal location and duplication events of the *GeBP* genes in nine species, including *B. distachyon* (**a**), *H. vulgare* (**b**), *O. sativa* ssp. *indica* (**c**), *O. sativa* ssp. *japonica* (**d**), *O. rufipogon* (**e**), *S. bicolor* (**f**), *S. italic* (**g**), *T. aestivum* (**h**), and *Z. mays* (**i**). The gray lines represent the whole-genome duplication (WGD)/segmental duplication events. The yellow curved boxes represent the different chromosomes. A high-resolution version of Figure 2 is provided in Figures S2–S10.

To better understand the expansion mechanism of the *GeBPs* paralogues in these species, the gene location and duplication pairs of each species were further analyzed. We mapped the *GeBP* gene sequences onto the genome and found that 125 GeBP genes of nine Gramineae species were unevenly distributed on the chromosomes (Figure 2). For example, in *O. sativa* ssp. *japonica* (Oj), there were three *GeBP* genes, both on chromosome 2 and chromosome 9, and two *GeBP* genes on chromosome 1 and chromosome 3, and only one *GeBP* gene was detected on chromosome 6 to chromosome 8. Meanwhile, 29 duplication gene pairs were identified in these Gramineae crops. All the duplication gene pairs were derived from the whole-genome duplication (WGD)/segmental duplication type (Table 1). Results showed that no duplication gene pair was found in Hv, but 4, 1, 3, 3, 4, 2, 5, and 7 duplication gene pairs were identified in Bd, Oi, Oj, Or, Sb, Si, Ta, and Zm, respectively. The numbers of duplication gene pairs varied greatly among those species, indicating that the gene expansion mechanism was different. Interestingly, the numbers and types of duplication gene pairs were the same between Oj and Or, which substantiated the previously reported that wild rice (Or) is an ancestor of cultivated rice [30].

Further analysis showed that the divergence time of all duplication gene pairs greatly varied from 0.19 to 47.98 million years (MYA) among these tested species (Table 1). For example, the divergence time of Bd duplication gene pairs ranged from 14.65 to 47.98 MYA and the Oi duplication gene pairs were similar to Oj and Or, ranging from 14.97 to 21.03 MYA. The Sb duplication gene pairs ranged from 0.25 to 26.80 MYA and the Si duplication gene pairs ranged more than 22 MYA. The Ta duplication gene pairs ranged from 0.19 to 5.46 MYA and the Zm duplication gene pairs ranged from 1.68 to 18.32 MYA. In addition to the *BdGeBP12/BdGeBP10* (the ratio of Ka/Ks = 0.7902), the Ka/Ks ratios of *GeBP* gene pairs were far less than 1, suggesting that purifying selection was accompanied by the evolution of *GeBP* genes (Table 1). These results showed that multiple duplication events played a role in the long-term process of *GeBP* gene expansion in the nine Gramineae crops. 

### 2.3. The Conserved Motif and Gene Structure Analysis 

**Figure 3:**
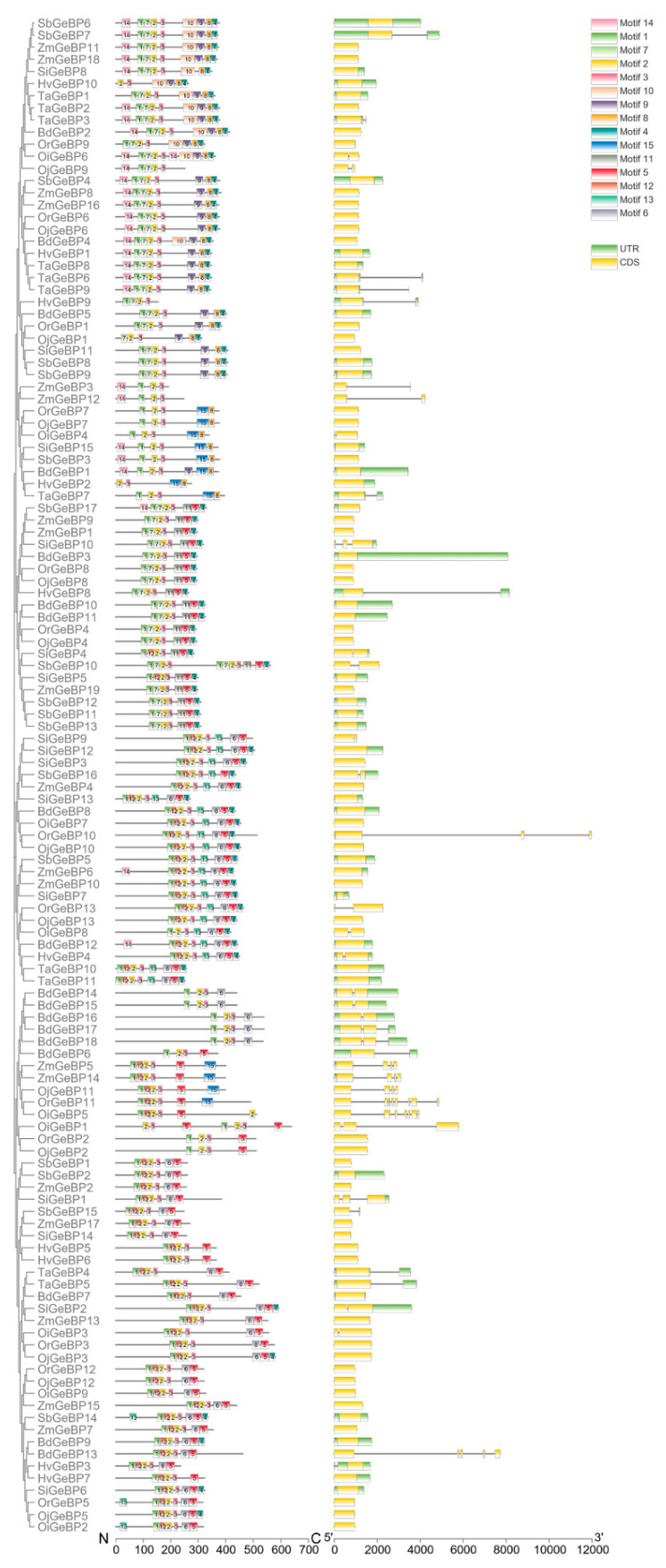
The protein conserved motif and gene structure analysis of the *GeBP* gene family in nine species. Motifs in the GeBP proteins were elucidated by TBtools. A phylogenetic tree was constructed using the MEGA 7.0 software. The different motifs are represented by different colored and numbered boxes. The different structures are marked in different colored boxes. The N and C represents the *N*-terminus and *C*-terminus ends of protein. The 5′ and 3′ represents the 5′ and 3′ ends of the gene.

To clarify the functional relationships of the *GeBP* gene family members during the revolution, conserved protein motifs and gene structures of 125 GeBP protein sequences were analyzed using the MEME program. The results showed a total of 15 conserved motifs in GeBPs (Figure 3). All the GeBP proteins contain motif1, motif2, and motif3, showing that these motifs might play crucial roles in the transcriptional regulation of their target genes. In addition, the motif distribution was similar among members of the same clade, but varied among different clades. These results suggested the diverse function of the GeBP proteins in Gramineae crops.

Meanwhile, we found that the structure of *GeBPs* had changed in the nine Gramineae crops, except for a fraction of genes. Most *GeBP* family members were found to be without intron, and few genes contained 2–8 exons. For example, *OiGeBP6* contained 2 exons, and *OrGeBP11* contained 8 exons. These results also suggested that the functions of the *GeBP* gene family were diverse and complicated in the plant kingdom. 

### 2.4. Expression Profiles of the GeBP Genes across Different Rice Tissues and Developmental Stages

**Figure 4:**
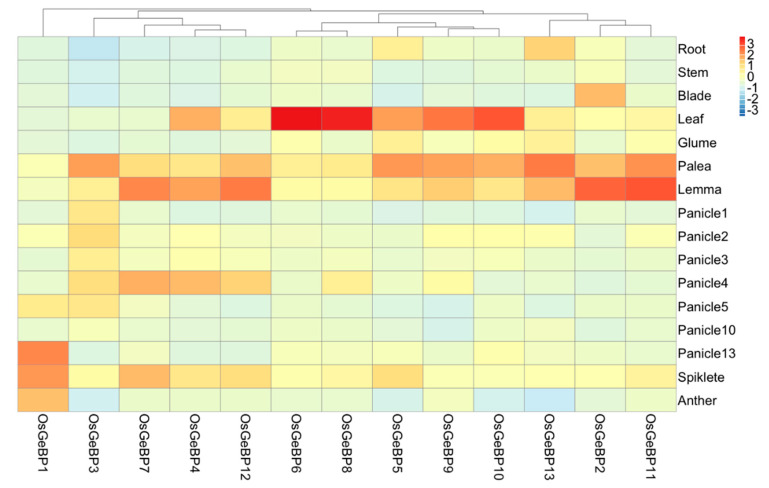
Expression profiles of the *GeBP* genes in the various rice tissues and developmental stages. A heatmap based on the data of qRT-PCR by the 2^∆ΔCT^ method indicates the clustering of 13 *OsGeBPs* in 16 tissues shown on the right. Gene names are shown on the bottom. The *OsGeBP* genes are clustered according to hierarchical clustering. All data were performed in three biological replicates and three technical replications.

To better understand the possible function of *GeBP*s in rice, we investigated the expression pattern in different tissues and developmental stages, including the root, stem, leaf, and reproductive organs by qRT-PCR. The results showed that 13 genes were expressed in many tissues at different developmental stages, with especially high levels of expression in the leaf, palea, and lemma (Figure 4). Through the expression profiles, 13 genes were mainly clustered into four groups based on hierarchical clustering analysis, revealing that four groups had a different function in rice growth and development. Interestingly, group I contains only one gene, *OsGeBP1*, which had the highest expression in the mature panicle, spikelet, and anther. Those results suggested that *OsGeBP1* might play an important role in the development of mature spikelets and anther in rice. In addition, group II comprising *OsGeBP3*, *OsGeBP7*, *OsGeBP4,* and *OsGeBP12* was also highly expressed in the young panicle and spikelet, apart from the palea and lemma, compared to that of group III and group IV.

### 2.5. Expression Profiles of the GeBP Genes in Rice under Various Hormonal Stresses

**Figure 5:**
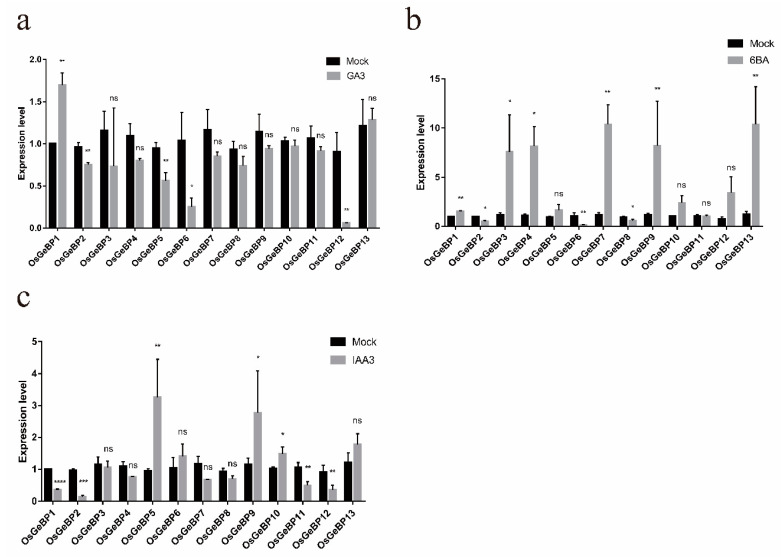
Expression profiles of the *GeBP* genes in rice under various hormonal stress. The expression level of 13 *OsGeBPs* was tested by qRT-PCR estimated by the 2^∆ΔCT^ method. The mock represents control, (**a**–**c**) represents treatment with gibberellin (GA3), cytokinin (6BA), and auxin (IAA), respectively. The error bars show the standard deviation of the three biological replicates. (Student’s *t*-test, ns, *p* > 0.05, * *p* ≤ 0.05, ** *p* ≤ 0.01, *** *p* ≤ 0.001, **** *p* ≤ 0.0001).

Phytohormones are essential for plant growth and development and play important roles in stress response. Previous studies have reported that several *GeBP* genes function in response to hormone treatments. Here, to testify whether Os*GeBPs* respond to various hormones, the seedlings were treated with GA3, 6BA, and IAA. One gene (*OsGeBP1*) was significantly induced and four genes (*OsGeBP2, OsGeBP5, OsGeBP6, and OsGeBP12*) were obviously decreased by GA3 treatments (Figure 5a). Six genes (*OsGeBP1, OsGeBP3*, *OsGeBP4*, *OsGeBP7, OsGeBP9,* and *OsGeBP13*) were induced and three genes (*OsGeBP2, OsGeBP6,* and *OsGeBP8*) were decreased by 6BA (Figure 5b). Three genes (*OsGeBP5*, *OsGeBP9*, and *OsGeBP10*) were induced and four genes (*OsGeBP1, OsGeBP2, OsGeBP11,* and *OsGeBP12*) were extremely decreased by IAA (Figure 5c), respectively. The transcript levels significantly increased or decreased under different treatments, implying the diverse functions of *OsGeBPs* in rice. Moreover, the results demonstrated that *OsGeBPs* had extremely strong responses to the cytokinin stimuli.

### 2.6. Expression Profiles of the GeBP Genes in Rice under Various Metal Ion Stresses

**Figure 6:**
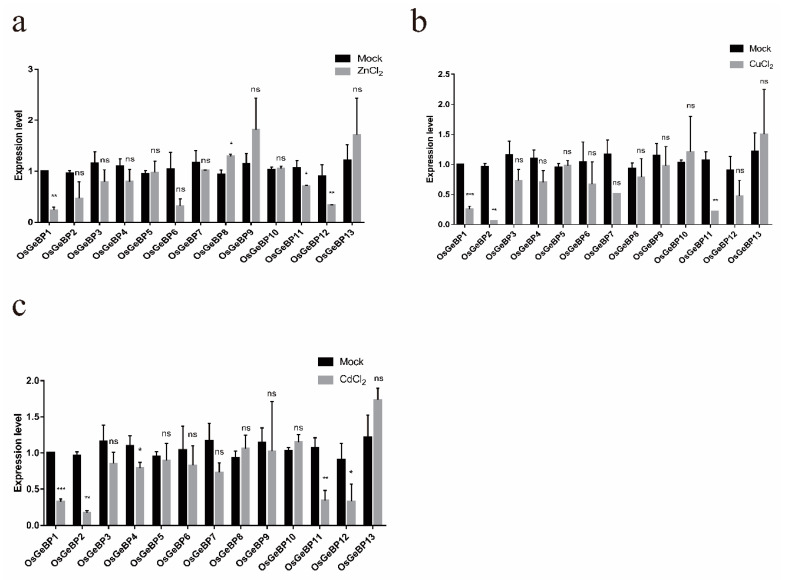
Expression profiles of the *GeBP* genes in rice under various metal ion stresses. The expression level of *OsGeBPs* was tested using qRT-PCR estimated by the 2^∆ΔCT^ method. The mock represents control, (**a**–**c**) represents the treatment with ZnCl_2_, CdCl_2_, and CuCl_2_, respectively. The error bars show the standard deviation of the three biological replicates. (Student’s *t*-test, ns, *p* > 0.05, * *p* ≤ 0.05,** *p* ≤ 0.01, *** *p* ≤ 0.001).

To assess the potential effects of metal ions on rice *GeBPs* expression during the development, the transcript levels were estimated under treatments with ZnCl_2_, CdCl_2_, and CuCl_2_. In our study, treatment with three metal ions significantly decreased the transcript levels of *OsGeBP1*, especially in response to CdCl_2_ and CuCl2 treatment (Figure 6a–c). In addition, *OsGeBP2, OsGeBP4*, *OsGeBP11*, and *OsGeBP12* were also downregulated in response to CdCl_2_ treatment (Figure 6c). Meanwhile, *OsGeBP2 and OsGeBP11* were downregulated in response to CuCl_2_ treatment (Figure 6b). Only *OsGeBP8* was gently induced by ZnCl_2_ treatment (Figure 6a). The above results demonstrated that *GeBP* genes may have played roles in response to various metal ion stresses in plant development. 

### 2.7. The Subcellular Localization of OsGeBP11 and OsGeBP12 in Rice

**Figure 7:**
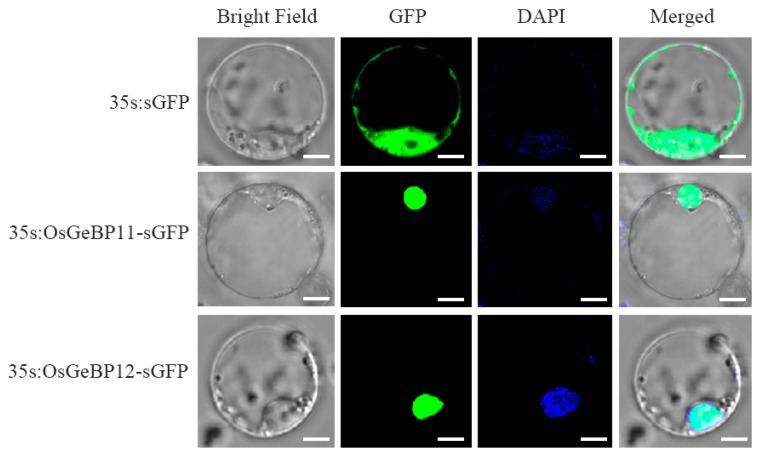
The subcellular localization of OsGeBP11 and OsGeBP12 in rice. The subcellular localization of OsGeBP11 and OsGeBP12 were detected through transient expression in the rice protoplast, the GFP signal represents the fusion proteins OsGeBP11-eGFP and OsGeBP11-eGFP; All data were performed in three independent experiments. The DAPI signal represents the nucleus, Scale bars: 5μm.

The localization of proteins is closely related to their function. In general, transcription factors are localized to the nucleus. To confirm whether OsGeBPs are also localized to the nucleus, we selected two genes, *OsGeBP11* and *OsGeBP12*, preferentially expressed in rice palea and lemma and constructed the 35s:OsGeBP11 and 35s:OsGeBP12 eGFP expression vectors. Both these vectors were transiently expressed in the rice protoplast, including 35S:eGFP were tested as the control (Figure 7). Results showed that the green fluorescence was characteristically observed in the nucleus of protoplast of the 35s:OsGeBP11-eGFP and 35s:OsGeBP12-eGFP, while the fluorescence was observed in the control. These results confirmed that the GeBP proteins were located in the nucleus, consistent with the previous studies on the other species [18]. Therefore, these data indirectly indicated that the OsGeBPs probably act as transcription factors to regulate the downstream genes during development and cope with various environments and stresses.

## 3. Materials and Methods

### 3.1. Plant Materials and Growth Conditions

The Nipponbare rice seeds (*O. sativa*. ssp. *japonica*) were germinated for 3 days in water placed in an incubator at 37 °C. Then, the sprouting seeds were transferred into a greenhouse with environmental conditions similar to those during summer in Wuhan. 

### 3.2. Hormone and Metal Ion Stress Treatments

For the hormone and metal ion stresses treatments, the seeds of Nipponbare were dehulled and sterilized with 0.15% HgCl_2_ solution for 10 min, rinsed 4 times with sterile distilled water. Next, these seeds were sown on the 3.5% Phytagel-solidified half-strength Murashige and Skoog medium supplemented with 100 μM GA3, 100μM N^6^-benzyladenine (6BA), 100 μM indole-3-acetic acid (IAA), 70 μM CdCl_2_, 65 μM CuCl_2_, and 0.5 mM ZnCl_2_, respectively, and grown for two weeks, in a controlled environment with a 16/8-h light/dark photoperiod at 26 °C, and 60% relative humidity. The control groups were maintained on the normal nutrient solution or medium. The samples were quickly frozen in liquid nitrogen and stored at −80 °C until use. The experimental procedure was replicated three times.

### 3.3. Genome-Wide Identification of the GeBP Genes in Gramineae Crops

The genome DNA, cDNA, and protein sequences of nine Gramineae crops (Brachypodium distachyon, Hordeum vulgare, Oryza. sativa ssp. Indica, Oryza. sativa ssp. japonica, Oryza rufipogon, Sorghum bicolor, Setaria italica, Triticum aestivum, and Zea mays) were obtained from the plant transcription factor database Plant Transcription (PlantTFDB v5.0) (http://planttfdb.gao-lab.org/index.php, accessed on 1 August 2021). The multiple alignments were performed using the MEGA software (v6.0) (choosing the “align by clustalW” option with default parameters) [31].

### 3.4. Phylogenetic Relationship Analyses

To construct the phylogenetic tree of the GeBP genes of the nine representatives of Gramineae crops, all the protein sequences of GeBPs were aligned using the ClustalW program (http://www.clustal.org/clustal2/, accessed on 15 February 2021) and edited with Jalview (http://www.jalview.org, accessed on 15 February 2021). Then, the phylogenetic tree was constructed by the neighbor-joining (NJ) method of the MEGA 7.0 (https://www.megasoftware.net/, accessed on 1 March 2021) software based on the following parameters: *p*-distance, pairwise deletion option, and 1000 bootstrap replications [32]. The classified and annotated GeBP protein sequences were visualized using the iTOL software (https://itol.embl.de/itol.cgi, accessed on 3 March 2021). 

### 3.5. Duplication Events, Orthologous Groups, Conserved Motifs, and Gene Structure Analyses

The duplicated gene pairs among the Gramineae crops were identified using the MCScanX with an E-value of 1 × e^−5^ in the BlastP search [33]. For the selective force analysis of a duplication gene pair, the nonsynonymous (Ka)/synonymous (Ks) substitution (Ka/Ks) rates were calculated using the TBtools [34]. The divergence time of each duplication gene pair was acquired using the formula T = Ks/ (2 × 9.1 × 10^−9^) × 10^−6^ (million years ago, MYA) [35].

To acknowledge the relationship of paralogous GeBPs in nine Gramineae, using the OrthoFinder v2.0 software, the phylogenetic tree of GeBPs was reconstructed depending on the result of orthologous groups using the STAG and STRIDE algorithms. The Tajima’s D values were calculated using the DnaSP 5.0 [36].

The intron-exon organizations were analyzed with the TBtools software [34]. The conserved motifs were detected through the MEME server v5.0.4, with a maximum number of 20 motifs and motif length between 5 wide and 200 wide amino acids [37]. The phylogenetic tree, motifs and gene structures were visualized using the TBtools software.

### 3.6. RNA Extraction, cDNA Synthesis, and Quantitative Real-Time PCR

The total RNA was extracted using the Trizol reagent (Invitrogen) (according to their instruction manuals) and RNase-free DNase I (Thermo Fermentas) treatment, to ensure that there is no contamination of DNA. The concentration of RNA was quantified using a Nanodrop 2000 spectrophotometer (Thermo Scientific, USA). Approximately 5 μg of the digested total RNA was reverse-transcribed into the first-strand cDNA with the M-MLV reverse transcriptase (Invitrogen) and random primer (Thermo Fermentas, MA, USA). The quantitative real-time PCR (qRT-PCR) was carried out on a CFX96 Touch ™ Real-Time PCR Detection System (Bio-Rad, Hercules, CA, USA). The specific qRT-PCR primers of 13 rice GeBP genes were designed using the Primer Premier 5 software and shown in Table S3. *Actin* was used as an internal control. The qRT-PCR was performed in three biological replicates and the technical replications were based on a previous study [38].

### 3.7. Transient Expression of the Enhanced Green Fluorescent Protein (eGFP) Constructs in the Rice Protoplast 

To construct the transient expression vectors 35s:OsGeBP11-eGFP and 35s:OsGeBP11-eGFP, the whole cDNA fragments of OsGeBP11 and OsGeBP12 were amplified from the Nipponbare cDNA and cloned into the HBT95-sGFP vector with a CMV35s promoter [39]. These constructs were further transformed into rice protoplasts by the polyethylene glycol 4000 (PEG4000)-mediated transformation method [40]. After incubation in the dark for 16 h, the GFP fluorescence was observed with a confocal laser-scanning microscope, with excitation at 488 nm and emission at 498 nm. (Leica TCS SP5). Three independent experiments were carried out. In each experiment, more than ten cells with positive signals were analyzed.

### 3.8. Statistical Analysis

All the data were analyzed using the GraphPad Prism 7.00 statistics program (https://www.graphpad.com/ accessed on 5 April 2021) and the means were compared by Student’s *t*-test. Each assay was performed in three biological replicates and technical replications. 

## 4. Discussion 

### 4.1. Characterization of the GeBP Genes in Gramineae Crops

The *GeBP* transcription factors have rarely been investigated in plants. In this study, a total of 125 *GeBP* genes were identified in nine Gramineae crops, including 18, 10, 9, 13, 13, 17, 15, 11, and 19 *GeBP* genes in *B. distachyon* (Bd)*, H. vulgare* (Hv)*, O. sativa* ssp. *indica* (Oi)*, O. sativa* ssp. *japonica* (Oj)*, O. rufipogon* (Or)*, S. bicolor* (Sb)*, S. italica* (Si)*, T. aestivum* (Ta)*,* and *Z. mays* (Zm), respectively (Figure S1). We found that *Z. mays* had the largest number of genes, containing 19 *GeBP* genes, consistent with the previous result that state that *Z. mays* underwent one specific WGDs compared to the other Gramineae plants [41]. Moreover, our results showed that the *GeBP* family genes could be classified into four clades by phylogenetic analysis and classification (Figure 1). The gene numbers varied greatly among each clade, indicating that the gene expansion mechanisms might be complicated. 

Gene duplication and divergence events are the main processes that multiply genetic material during evolution and selection [42]. To further investigate the expansion mechanism in these species, the gene location and duplication pairs of each species were analyzed. In our study, we found that all of the *GeBP* genes were unevenly distributed on the chromosomes (Figure 2). Additionally, 29 duplication gene pairs were identified, all of which were derived from the WGD/segmental duplication type (Table 1). These results indicated that the WGD/segmental duplication type appears to have served as the most important driving force throughout the long period of Gramineae crops gene evolution. The numbers of duplication gene pairs varied greatly among these species, such as no duplication gene pairs in *H. vulgare* (Hv), and seven duplication gene pairs were found in *Z. mays* (Zm). The above results indicated that the gene expansion mechanism was different in these Gramineae crops. Further analysis showed that the divergence time of all duplication gene pairs varied greatly among these tested species, ranging from 0.1898 to 47.9755 Mya. The ratio of non-synonymous versus synonymous substitutions (Ka/Ks) is an indicator of the history of selection acting on a gene or gene region [43]. In our study, the Ka/Ks values of all the identified duplicate pairs were less than 1.0. These results suggested that multiple duplication events played essential roles in the gene expansion of Gramineae crop genomes during the long-term evolutionary process.

To better understand the functional relationships of the *GeBP* gene family, the protein conserved motif and gene structure were further analyzed using the MEME program. A total of 15 conserved motifs were identified among 125 GeBP proteins, and divided into different clusters (Figure 3). The result showed that different clusters had a great difference in members and distribution of the conserved motif. Interestingly, all GeBP proteins had motif1, motif2, and motif3, showing that these motifs may play crucial roles in transcript regulation of target genes expression during the evolutionary process. Moreover, the gene structure of *GeBPs* was quite different in the nine Gramineae crops, indicating that the function of the *GeBP* genes family was different in the plants. 

### 4.2. Expression Profiles of the GeBP Genes in Gramineae Crops

As a sessile organism, Gramineae crops have to confront complicated environments. The transcription factors have been suggested as key regulators to modulate gene expression, responding to various environmental stress [44]. Previously, studies showed that the *GeBP* genes family play important roles in plant growth and development, as well as response to hormone and ion, implying that the *GeBP* genes are involved in various hormonal pathways [7,18,26]. We analyzed the expression profiles of the *GeBP* genes in rice at different developmental stages and in different tissues by quantitative real-time PCR. The results showed that 13 genes were expressed in many tissues at different development stages, and especially high expression in leaf, palea, and lemma (Figure 5). This supported the previous report that *GeBP* genes can regulate the development of the plant epidermis cells [25].

Moreover, the *GeBP* genes were predicted to play a role in various hormonal pathways [16]. Here, we found that the rice *GeBP* genes respond differently to GA3, 6BA, and IAA, which suggest that they had functional differentiation (Figure 5). For example, the transcription level of *OsGeBP1* was upregulated and the transcription levels of *OsGeBP2, OsGeBP5, OsGeBP6,* and *OsGeBP12* were downregulated by the application of exogenous GAs. These results were consistent with the previous report that the expression of the *GeBP* genes family was regulated by GA hormones. However, the mechanism by which the GAs regulates the expression of *GeBP* genes is still elusive. Additionally, the expressions of *OsGeBP1, OsGeBP3*, *OsGeBP4*, *OsGeBP7*, *OsGeBP9*, and *OsGeBP13* were induced by cytokinin, which is also consistent with the report that GeBP/GPL play a redundant role in the cytokinin hormone pathway [26]. Meanwhile, we showed that auxin can also induce the expression of *GeBP* genes, indicating that auxin might promote the formation of trichomes by regulating the expression of *GeBP* family genes, but the mechanism still needs further study.

Previous studies have characterized a transcription factor, *GeBP-LIKE 4 (GPL4)*, which was induced rapidly in the root tips in response to cadmium (Cd), and functioned as an inhibitor of root growth in *Arabidopsis* [21]. In our study, a small number of the *GeBP* genes were induced by heavy metal ions in the compounds, ZnCl_2_, CdCl_2_, and CuCl_2_, which implied that the *GeBP* genes played crucial roles in response to various metal ion stresses in plant development. Interestingly, we found that *OsGeBP1* had significant responses to three hormones and three metal ions. The transcript levels of *OsGeBP1* were significantly induced by gibberellin and cytokinin while decreased by auxin. Meanwhile, the transcript levels of *OsGeBP1* were significantly decreased in response to three metal ion stresses. These results suggest that *OsGeBP1* may play an important role in responding to environmental changes and stresses. However, the mechanism by which the *GeBP* genes respond to heavy metals needs further investigation. Subsequently, we confirmed that the subcellular localizations of the OsGeBP11 and OsGeBP12 were observed in the nucleus, suggesting that OsGeBPs functioned as transcription factors. Taken together, these results showed that *OsGeBP* genes can respond to various stresses or hormones to further regulate the downstream target genes in rice, suggesting that the *GeBP* family might also be involved in various regulatory networks to endure the complicated and unfavorable environments. Despite the biological functions of *GeBP* remaining elusive, our study has been able to present the fundamental data for the further exploration of GeBP in plants. 

## 5. Conclusion 

In our study, the comprehensive analysis of nine Gramineae crops of the *GeBP* family identified 125 genes that were classified into four clades, and divided into nine orthologous groups (OGs). The bioinformatic analyses and expression profiles indicated that there were different expansion mechanisms and there might be different functions of the *GeBP* gene family among these tested species, but further experimental work will be required to confirm this. Thus, these results provided a foundation for further understanding of the biological roles of the individual *GeBP* gene in Gramineae crops. 

## Figures and Tables

**Table 1:** Ka/Ks values and divergence time of all duplication gene pairs.

Seq_1	Seq_2	Ka	Ks	Ka/Ks	Duplication Type	Date (MYA)	Purifying Selection
BdGeBP10	BdGeBP3	0.2840	0.5749	0.4940	WGD or segmental duplication	15.61	YES
BdGeBP12	BdGeBP10	0.8732	1.1050	0.7902	WGD or segmental duplication	47.98	YES
BdGeBP12	BdGeBP8	0.3481	0.7242	0.4807	WGD or segmental duplication	19.13	YES
BdGeBP2	BdGeBP4	0.2667	0.5949	0.4483	WGD or segmental duplication	14.65	YES
OiGeBP7	OiGeBP8	0.3827	0.7380	0.5185	WGD or segmental duplication	21.03	YES
OjGeBP4	OjGeBP8	0.3516	0.5702	0.6166	WGD or segmental duplication	19.32	YES
OjGeBP6	OjGeBP9	0.2728	0.5102	0.5346	WGD or segmental duplication	14.99	YES
OjGeBP10	OjGeBP13	0.3549	0.6963	0.5097	WGD or segmental duplication	19.50	YES
OrGeBP6	OrGeBP9	0.2853	0.6970	0.4093	WGD or segmental duplication	15.67	YES
OrGeBP4	OrGeBP8	0.3498	0.5667	0.6172	WGD or segmental duplication	19.22	YES
OrGeBP10	OrGeBP13	0.3429	0.7032	0.4876	WGD or segmental duplication	18.84	YES
SbGeBP17	SbGeBP10	0.4878	1.0186	0.4789	WGD or segmental duplication	26.80	YES
SbGeBP4	SbGeBP6	0.3067	0.8060	0.3806	WGD or segmental duplication	16.85	YES
SbGeBP5	SbGeBP16	0.3966	0.8534	0.4647	WGD or segmental duplication	21.79	YES
SbGeBP8	SbGeBP9	0.0045	0.0359	0.1244	WGD or segmental duplication	0.25	YES
SiGeBP10	SiGeBP4	0.4122	0.6411	0.6430	WGD or segmental duplication	22.65	YES
SiGeBP12	SiGeBP7	0.4039	0.7101	0.5687	WGD or segmental duplication	22.19	YES
TaGeBP10	TaGeBP11	0.0035	0.0843	0.0410	WGD or segmental duplication	0.19	YES
TaGeBP1	TaGeBP2	0.0739	0.1727	0.4280	WGD or segmental duplication	4.06	YES
TaGeBP1	TaGeBP3	0.0758	0.1790	0.4233	WGD or segmental duplication	4.16	YES
TaGeBP2	TaGeBP3	0.0107	0.0813	0.1319	WGD or segmental duplication	0.59	YES
TaGeBP4	TaGeBP5	0.0993	0.1508	0.6587	WGD or segmental duplication	5.46	YES
ZmGeBP10	ZmGeBP6	0.0599	0.2385	0.2510	WGD or segmental duplication	3.29	YES
ZmGeBP11	ZmGeBP16	0.3334	0.6935	0.4808	WGD or segmental duplication	18.32	YES
ZmGeBP11	ZmGeBP8	0.3210	0.8094	0.3966	WGD or segmental duplication	17.64	YES
ZmGeBP12	ZmGeBP3	0.0340	0.1367	0.2487	WGD or segmental duplication	1.87	YES
ZmGeBP18	ZmGeBP11	0.0306	0.1401	0.2183	WGD or segmental duplication	1.68	YES
ZmGeBP1	ZmGeBP9	0.0418	0.1690	0.2474	WGD or segmental duplication	2.30	YES
ZmGeBP8	ZmGeBP16	0.0377	0.1678	0.2248	WGD or segmental duplication	2.07	YES

For selective force analysis of a duplication gene pair, the Ka/Ks rates were calculated using TBtools. The divergence time of each duplication gene pair was acquired using the formula T = Ks/ (2 × 9.1 × 10^−9^) × 10^−6^. The MYA represents million years ago, the WGD represents whole genome duplication.

## Data Availability

The sequences of genes and proteins of nine representatives of Gramineae crops (B. distachyon, H. vulgare, O. sativa ssp. indica, O. sativa ssp. japonica, O. rufipogon, S. bicolor, S. italica, T. aestivum, and Z. mays) mentioned in our study are available for download from the public database mentioned above. The GenBank accession numbers of GeBPs were listed in Table S1.

## Supplementary Materials

The following are available online at https://www.mdpi.com/article/10.3390/ijms22168758/s1.

## Abbreviations

GeBP	GLABROUS1 enhancer-binding protein
TF	Transcription factor
GA	Gibberellin
CK	Cytokinin
cDNA	Complementary DNA
NJ	Neighbor-joining
Ka/Ks	nonsynonymous (Ka) /Synonymous (Ks) substitution
qRT-PCR	Quantitative real-time PCR
Bd	*Brachypodium distachyon*
Hv	*Hordeum vulgare*
Oi	*Oryza. sativa* ssp.*indica*
Oj	*Oryza. sativa* ssp. *japonica*
Or	*Oryza rufipogon*
Sb	*Sorghum bicolor*
Si	*Setaria italica*
Ta	*Triticum aestivum*
Zm	*Zea mays*
WGD	Whole genome duplication
MYA	Million years
OGs	Orthologous groups
