# Supplementary material for: Genome-Wide Identification, Expansion Mechanism and Expression Profiling Analysis of GLABROUS1 Enhancer-Binding Protein (GeBP) Gene Family in Gramineae Crops
Source: Int J Mol Sci. 2021 Aug 15;22(16):8758. doi: 10.3390/ijms22168758 (PMC8395763; doi:10.3390/ijms22168758)
Supplement: Supplementary file 1 [file ijms-22-08758-s001.zip › ijms-1290330-supplementary.pdf]

International Journal of
Molecular Sciences

Supplementary file

Genome-Wide Identification, Expansion Mechanism and Expression Profiling Analysis of *GLABROUS1 Enhancer-Binding Protein (GeBP)* Gene Family in Gramineae Crops

Jishuai Huang [1], Qiannan Zhang [1], Yurong He [1], Wei Liu [2], Yanghong Xu [3], Kejia Liu [1], Fengjun Xian [1], Junde Li [1] and Jun Hu [1,*]

1 State Key Laboratory of Hybrid Rice; Engineering Research Center for Plant Biotechnology and Germplasm Utilization of Ministry of Education; College of Life Sciences, Wuhan University, Wuhan, 430072, Hubei, China, huangjishuai28@whu.edu.cn (J.H.); qiannanzhang813@whu.edu.cn (Q.Z.); heyurong@whu.edu.cn (Y.H.); 2017300030057@whu.edu.cn (K.L.); 2019202040064@whu.edu.cn (F.X.); 2016301060020@whu.edu.cn (J.L.)
2 School of Biological Science, University of Bristol, Bristol BS8 1TQ, UK, gz19065@bristol.ac.up
3 Institute of Neuroscience, State Key Laboratory of Neuroscience, Center for Excellence in Brain Science and Intelligence Technology, Chinese Academy of Sciences, 200000, Shanghai, China, xuyh@ion.ac.cn
* Correspondence: junhu@whu.edu.cn

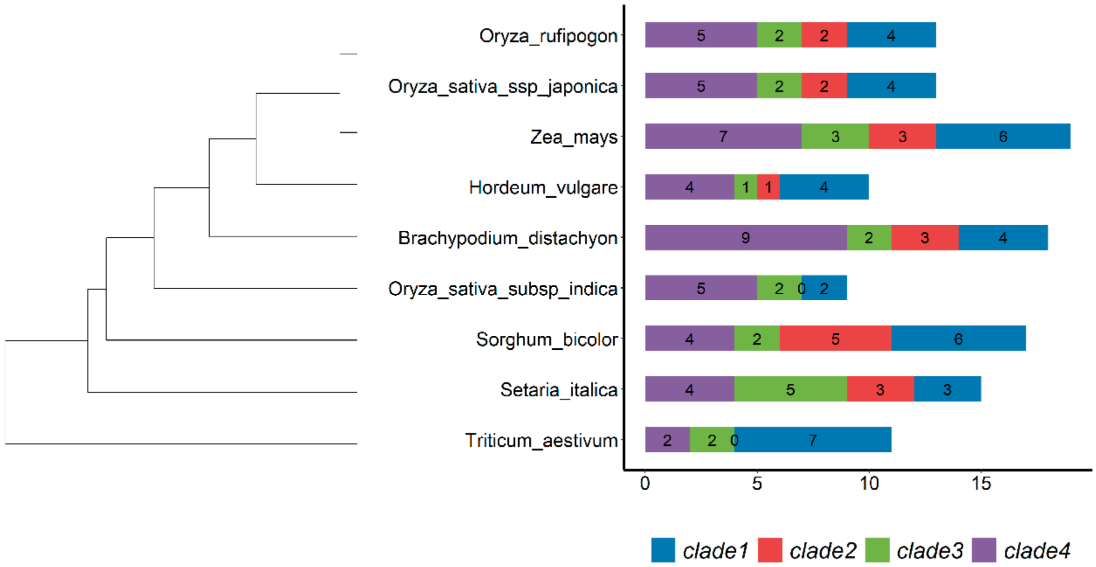

Figure S1. The GeBP gene numbers of four clades among nine species, including O. rufipogon, O. sativa ssp. japonica, Z. mays, H. vulgare, B. distachyon, O. sativa ssp. indica, S. bicolor, S. italic, T. aestivum. Different colors of rectangle represent different clade. The number on the rectangle represents the number of genes.

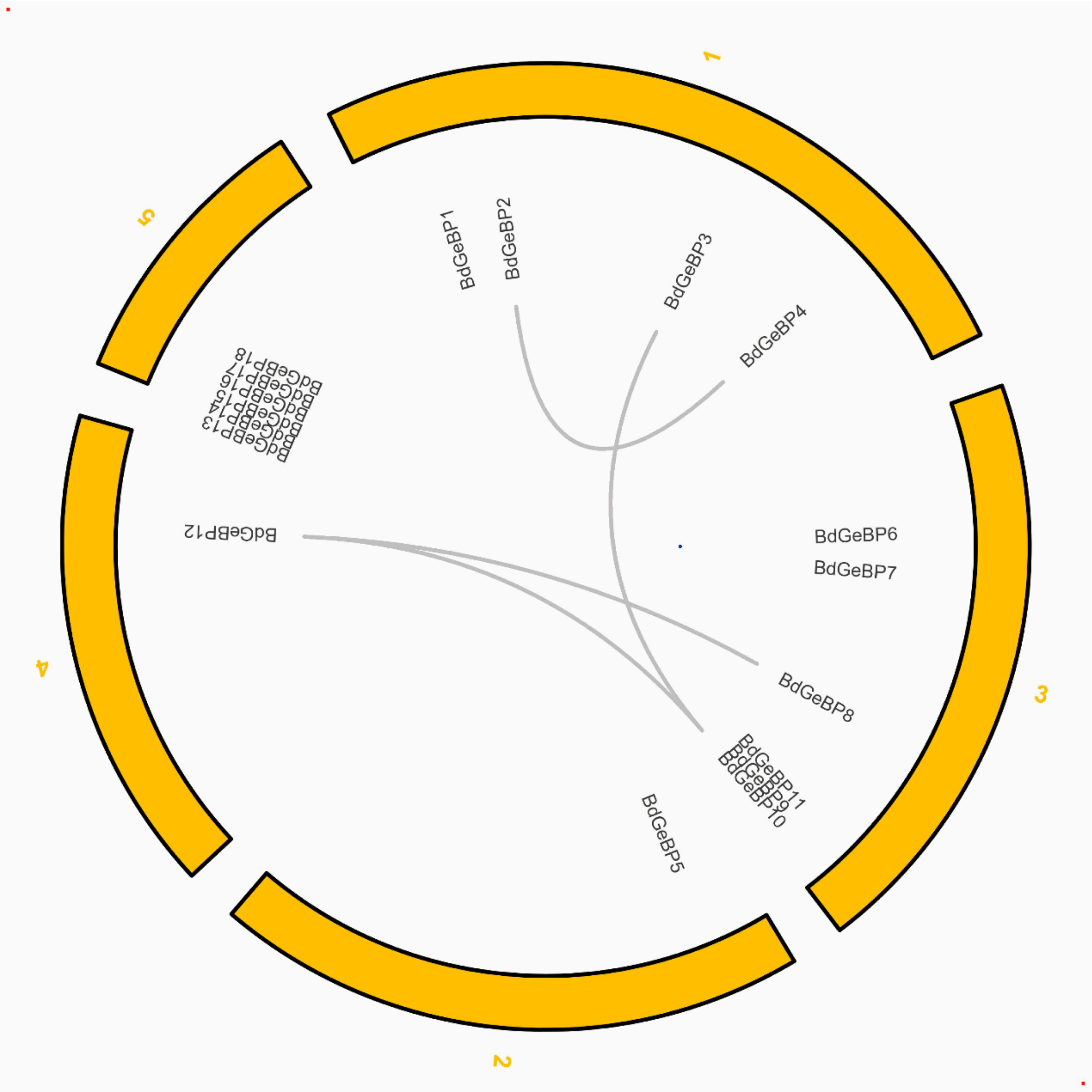

Figure S2. The chromosome location and duplication events of GeBP genes in *B. distachyon*.

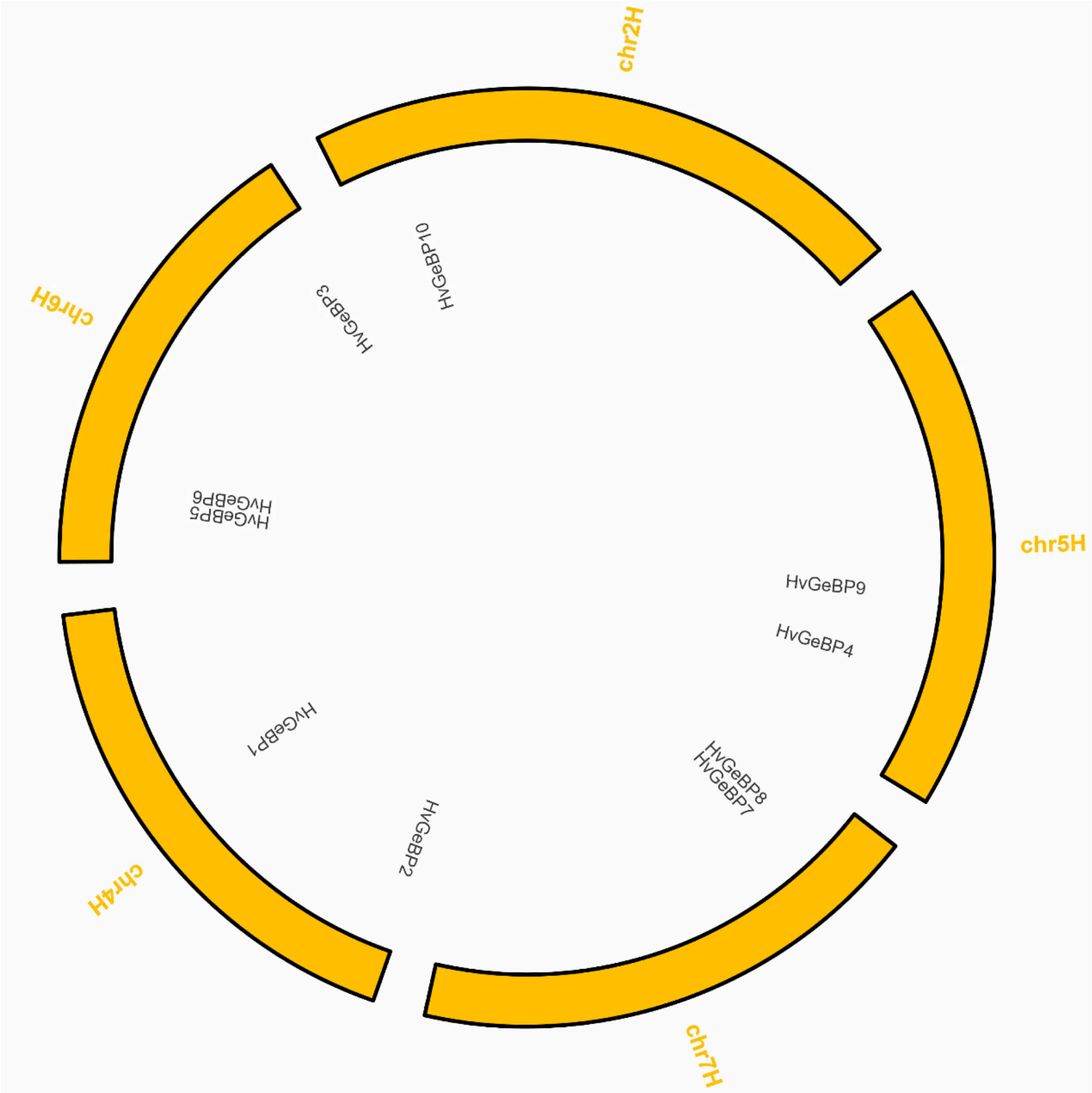

Figure S3. The chromosome location and duplication events of GeBP genes in *H. vulgare*.

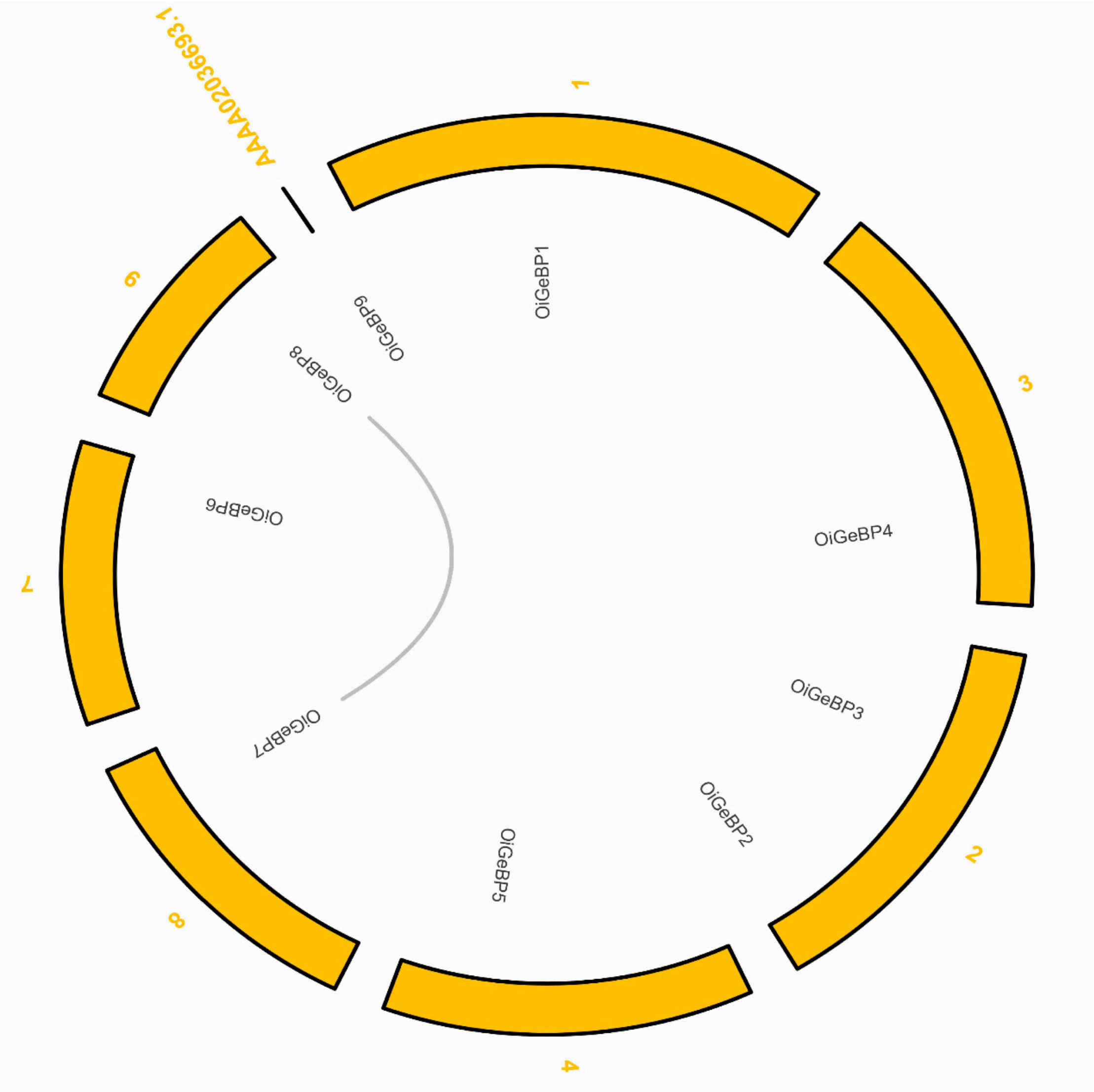

Figure S4. The chromosome location and duplication events of GeBP genes in *O. sativa* ssp. *indica.*

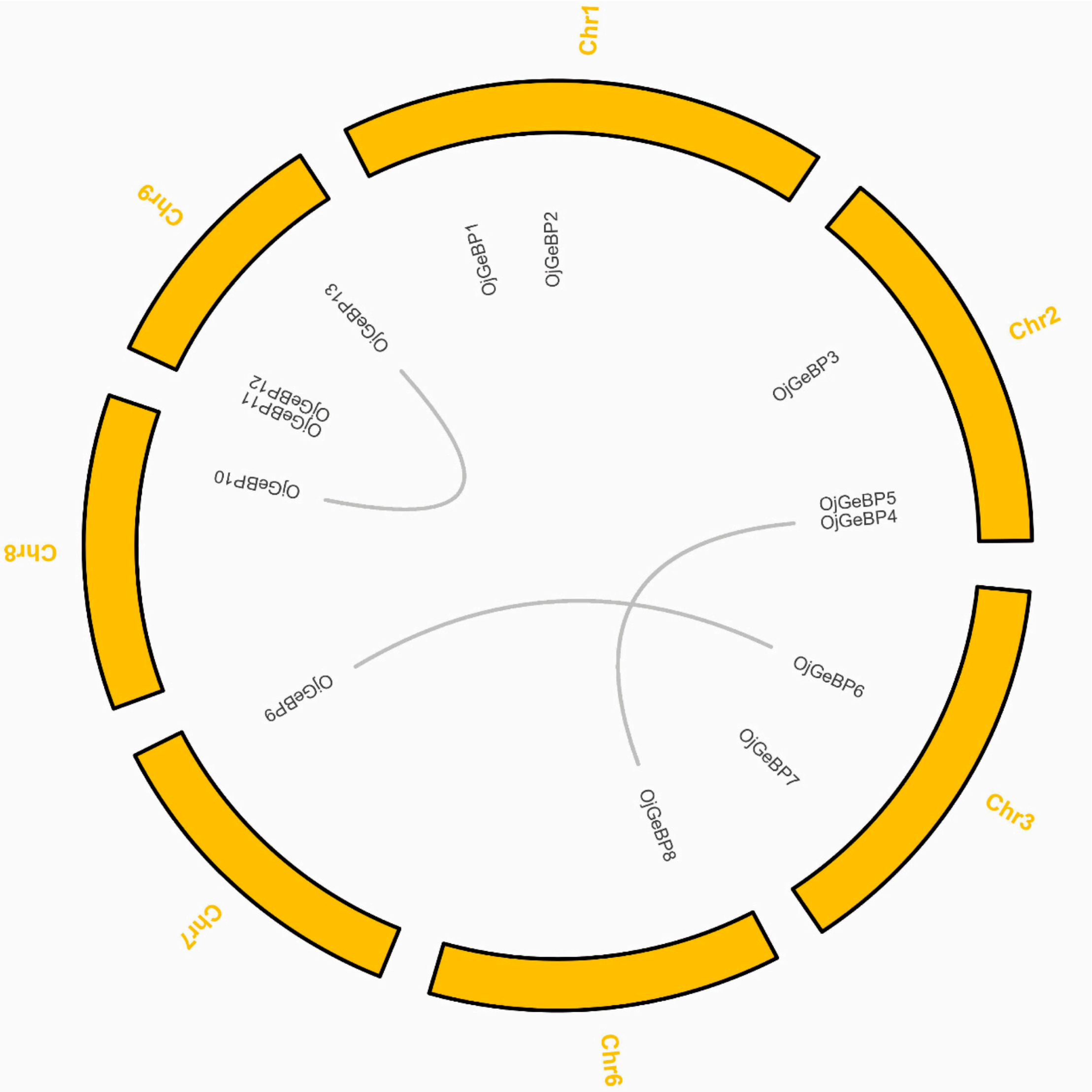

Figure S5. The chromosome location and duplication events of GeBP genes in *O. sativa ssp. japonica.*

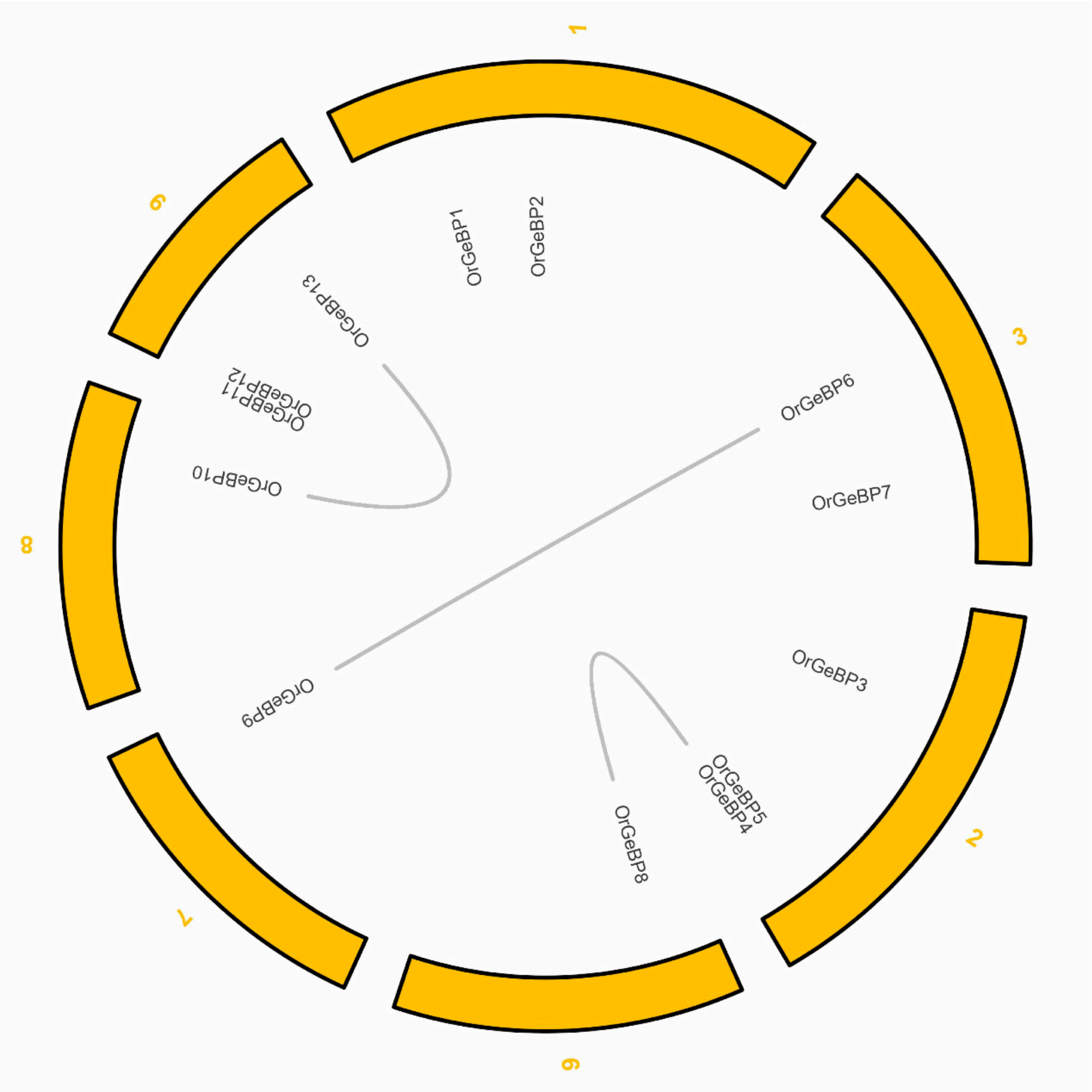

Figure S6. The chromosome location and duplication events of GeBP genes in *O. rufipogon*.

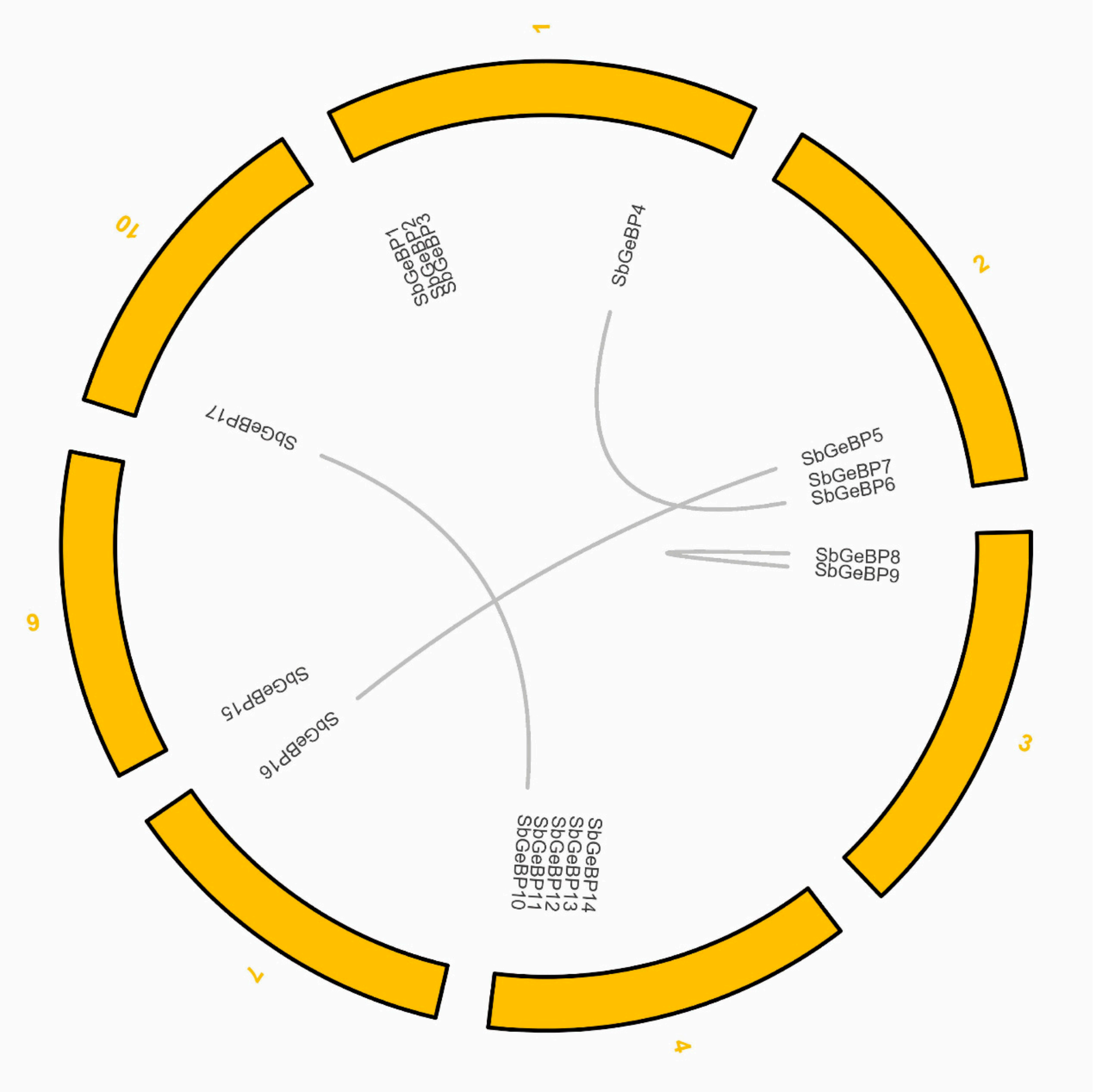

Figure S7. The chromosome location and duplication events of GeBP genes in *S. bicolor*.

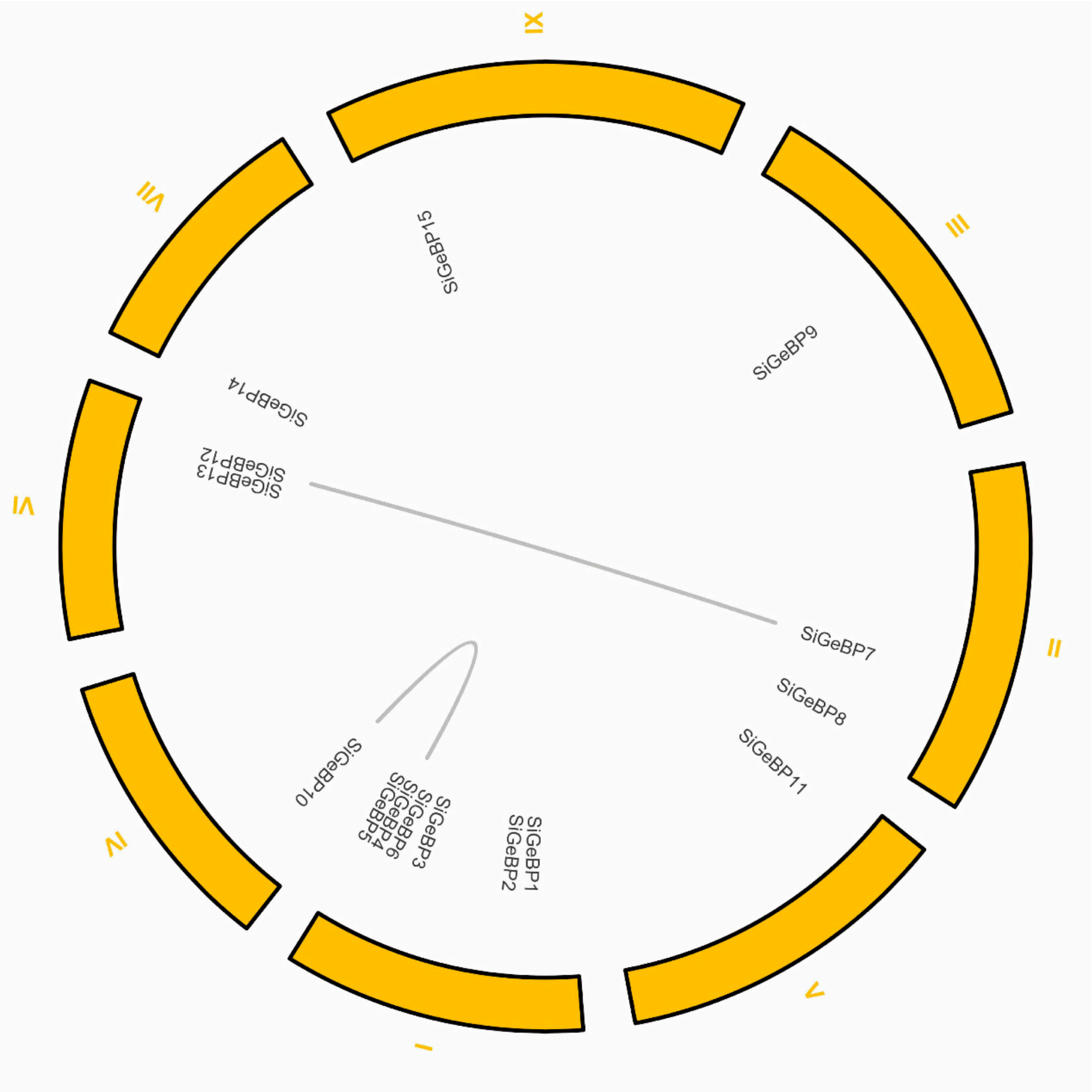

Figure S8. The chromosome location and duplication events of GeBP genes in *S. italic*.

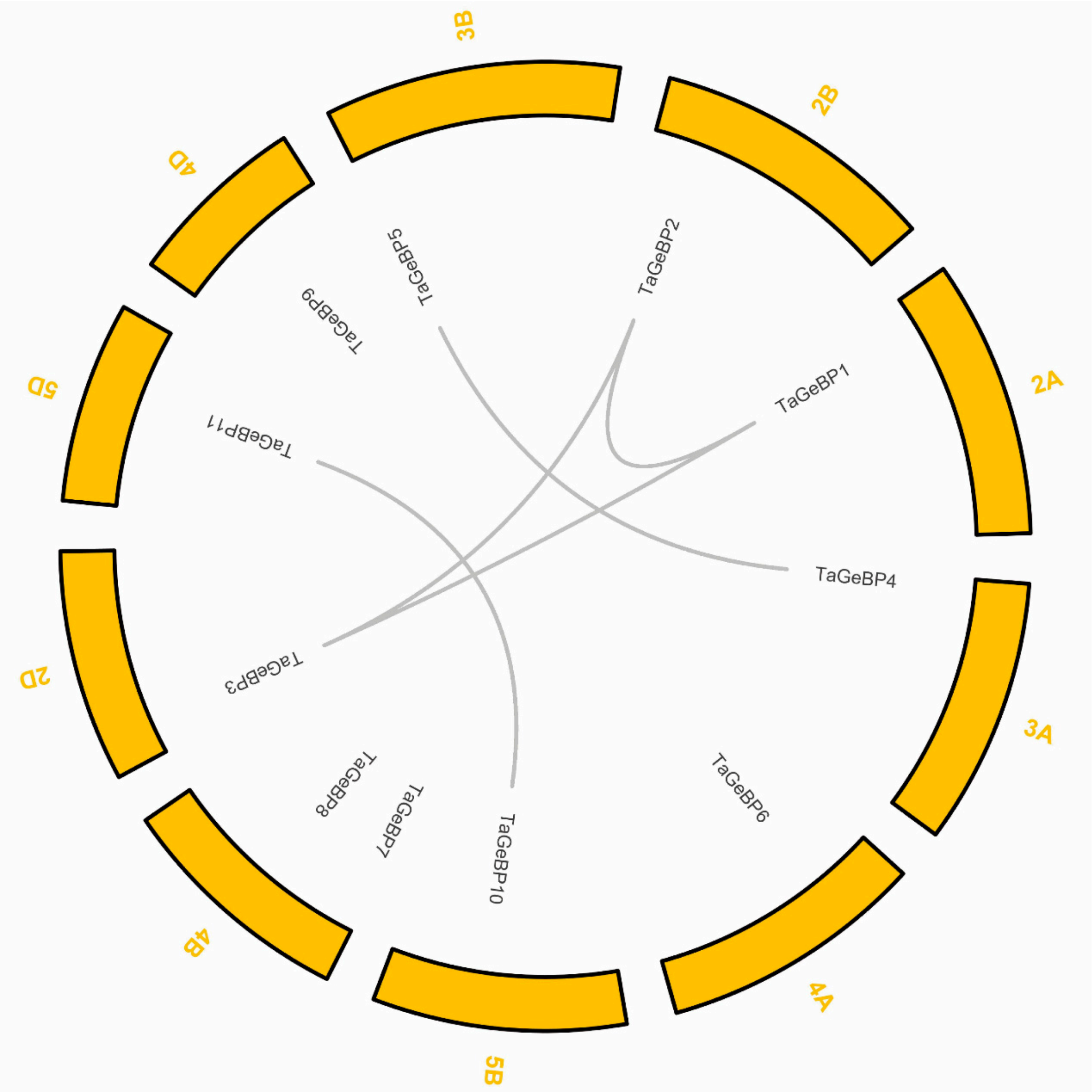

Figure S9. The chromosome location and duplication events of GeBP genes in *T. aestivum*.

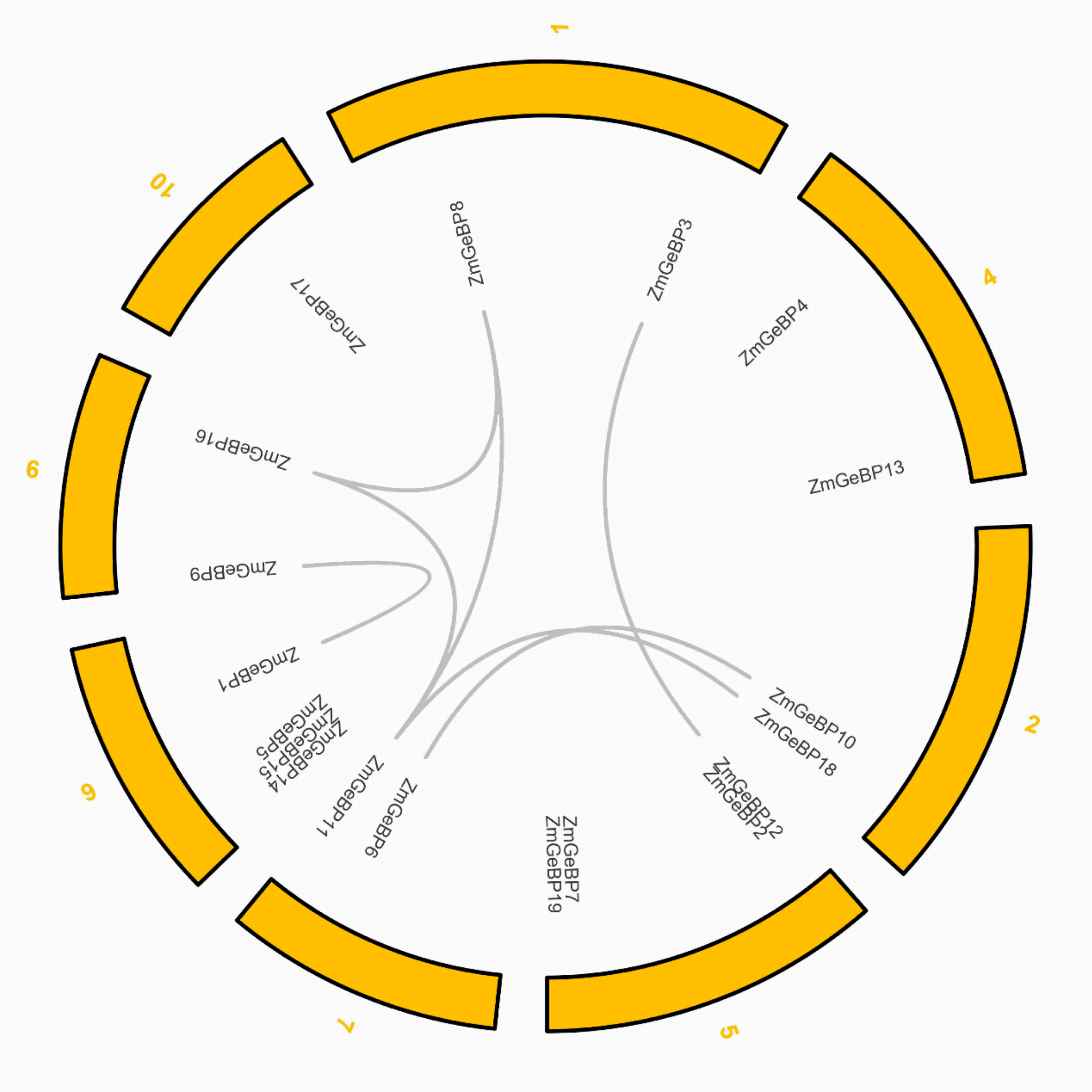

Figure S10. The chromosome location and duplication events of GeBP genes in *Z. mays*.

>O. sativa ssp. japonica OsGeBP11 complementary DNA sequence

ATGGCGCCCACTCTCCCCGTCGTCCCCGCCCACGGGGAGGACAAGAAGAA-
GAAGAAGAAGAA-
GAAGAAGCCGCTCAAGCCTTCGCAGGAGGAGGAGGAGGCCCTGCCGCTGCCGC
TGCCGCCGCCGGATCGCAAGAGGAAGAAGGCATCGGAGCCGGTCAACTCGCCG-
GAGA-
GAGCCAAGAAGAAGAAGACGGCGACGCCCCACGAGCCGCCATCGGCCAAGCA
GCAGAAAAGACCGCTCCCCTTCCAGCGCACGTGGAGCCCCAACGAC-
GAGGTCCTCATCCTT-
GAGGCCATGGCGGCGCATCGCCAGGAGCACGGCAAGGTACCTACCGCCGCGGA
GCTCTTCCCCGTCCTCAACGGACGCCTCGACAGGAAGCGCCTCACCTATAAGAA-
GCTCGCCGACAAGTTGCGCACCTTCATGCGCCGCCATGGCAGAGACGCCAAGA
ATGGCCCGCCCACCCAAGCGCACGACCGCCGCCTCTACGACCTCTCCAG-
GAATGTCTGGGTGTCTCAAACTCAACCGCCAAACCTAAGCGCCAACGCCAATTC
AAACATCGCGGGCGGGCAACCCAACCAGCATGATGCCATGCCCACCG-
CAGGCAAGGCGTTCGACAAAATGCGCGACTCCTACCCCAACCTCACCCAAGCG
CTGTTGCTTCTTGTGGGTACGGACCTGGAGAAAGCGCTCACTGCCATTGAC-
GAAAC-
CAAGGCCCAGGCGCTGGACTTGAAGGTCAGCAACCTCAAGAAGGAGCTGTCTG

AGGCTGTCATGGAATCGGCAACGATTCAAAGCACAGAAAGTTCTAAAATAC-
CGTGCTTTCCATCTACCAAGCTTCAACCAGAGTTTGGTGCAGAAATTGAGAAGA
ACTTTCAGCTAGAGCATTTGGATGAAATGAAAGGCACCCAAGTGAAATTGG-
CAA-
GAATGGAACAAGAAATATTAGAACTGAAACAAAATTTTTAGCTTTTCAGTCTC
AGCAAATGGCTGATAGCAAGCAACAACACGACAAGTCATCAGCGAAGGG-
TATAA-
TATGTGAATCTTCTGAGAGTGGTCTTCGTTCAATTGTAGCTGACAATAATATTTTA
TGCAATACTTTGCAAAAGGAGATGGTGGTACAACAGAAATTGTCTTGCGGAA-
GAC-
CAAGGAGGTTACTTCTAAGCATCGACATCCTCAGAAGCTGGTTGTCTTTCCTTTTT
GA

>O. sativa ssp. japonica OsGeBP12 complementary DNA sequence

ATGAGCTCATGGTGGTCGTGGCGGAGCCTCTTCTCCTCCTTGGCCAAC-
GCCAACGGAGGGGG-
CAGCAATGCCGATGCTTCCTCCGGATCCGGCACCTCCTCCTCCCCTCCCGTCCAT
GAAGCTCAACAAGCAGCAGCTCGCCGGCGAAGCGCTCGCACCAAGAA-
GCCTCCCGAGGAA-
GAAGCAGCAGGATCGCAGCCGCAGCCCAAGACTCGTCCTTCACCGGCGTCCAA
GGCGTCCAAGGCGAAGGTGCTGCTGCTGCTCGGCGACGGCGAGCCCAAGAA-
GAA-
GCCAGCCCCGAACCCGACCCCGACTCAGAAGCGTAGCAACAAGAGAAAGCGGT
CGTGGTCCAGGGCCGACGAGCTCAGGATTCTGGAGGCCATGGCCAACCAC-
GCCAAC-
GCCCATGGCGGCGCTCTGCCGGAGGCCTCCGATCTCTTTGCCGCCCTTGCCAGCA
GCCTCGAGAGAGGAGATGCCGACCTGCCCAAGCTCGCCGACAAGGTCCACAA-
GCTCAAGA-
GATGGTATGACAATGCACGCCTGCCGCAGCGCTGCCCGACTGACGATGACGACG
ATACGCGTCGATTGTTCCAGCTATGTGGGAAGGTTT-
GGGGTCCCCCTTCAACTGTGCTGCG-
CACGAGCCCACGCCAACGCCACAAGGTAGTAGGAGTACTTGTCCAAGGAAATG
GTGCCAATCCCCAGCCGGCAGCAGCACTCAAGGTCAAGGAGAAGAGAGTCAG-
GAGGGAG-
TTATCTGAGCTTTATGTCCTGTATCCTTGTCTCGCCCAGGAGGTCAAGGCGCATG
CTAACGAATACGGCGAGCTCATCGGAACAGCCTTTCAGTTCATCGGCGATGAC-
GAA-
GCTCGATGTTACGACGACAGGTACAGGAAGATGTTGGTGGACAAGCTTAACATG
AAGAAGGAACATGCCGACGTAACAAAGTCCCTGCTGTGCACTCTTGCAGGC-
TACATAAATTAG

>nucleotide construct for transient expression vector of 35s:OsGeBP11-eGFP

cgatgataagctgtcaaacatgagaattcg-
taatcatggtcatagctgtttcctgtgtgaaattgttatccgctcacaattccacacaacatacgagccggaagcataaagtgtaaa
gcctggggtgcctaatgagtgagctaactcacattaattgcgttgcgctcac-
tgcccgctttccagtcgggaaacctgtcgtgccagctgcattaatgaatcggccaacgcgcggggagaggcggtttgcgtattg
ggcgctcttccgcttcctcgctcac-
tgactcgctgcgctcggtcgttcggctgcggcgagcggtatcagctcactcaaaggcggtaatacggttatccacagaatcagg
ggataacgcaggaaagaacatgtgagcaaaaggccagcaaaaggccaggaaccg-
taaaaaggccgcgttgctggcgtttttccataggctccgcccccctgacgagcatcacaaaaatcgacgctcaagtcagaggtg
gcgaaacccgacaggactataaagataccaggcgtttccccctggaa-
gctccctcgtgcgctctcctgttccgaccctgccgcttaccggatacctgtccgcctttctcccttcgggaagcgtggcgctttctcat
agctcacgctgtaggtatctcagttcggtgtaggtcgttcgctccaagctgggctgtgtg-
cacgaacccccсgttcagcccgaccgctgcgccttatccggtaactatcgtcttgagtccaacccggtaagacacgacttatcgcc

actggcagcagccactggtaacaggattagcagagcgaggtatgtaggcggtgctacagag-
ttcttgaagtggtggcctaactacggctacactagaaggacagtatttggtatctgcgctctgctgaagccagttaccttcggaaa
aagagttggtagctcttgatccggcaaacaaaccaccgctggtagcggtggtttttttgttt-
gcaagcagcagattacgcgcagaaaaaaaggatctcaagaagatcctttgatcttttctacggggtctgacgctcagtggaacg
aaaactcacgttaagggattttggtcatgagattatcaaaaaggatcttcaccta-
gatccttttaaattaaaaatgaagttttaaatcaatctaaagtatatatgagtaaacttggtctgacagttaccaatgcttaatcagtg
aggcacctatctcagcgatctgtctatttcgttcatccatagttgcctgactccccgtcgtg-
tagataactacgatacgggagggcttaccatctggccccagtgctgcaatgataccgcgagacccacgctcaccggctccagat
ttatcagcaataaaccagccagccggaagggccgagcgcagaagtggtcctg-
caactttatccgcctccatccagtctattaattgttgccgggaagctagagtaagtagttcgccagttaatagtttgcgcaacgttgt
tgccattgctacaggcatcgtggtgtcacgctcgtcgtttgg-
tatggcttcattcagctccggttcccaacgatcaaggcgagttacatgatcccccatgttgtgcaaaaaagcggttagctccttcgg
tcctccgatcgttgtcagaagtaagttggccgcagtgttatcactcatggttatggcagcac-
tgcataattctcttactgtcatgccatccgtaagatgcttttctgtgactggtgagtactcaaccaagtcattctgagaatagtgtatg
cggcgaccgagttgctcttgcccggcgtcaatacgggataatac-
cgcgccacatagcagaactttaaaagtgctcatcattggaaaacgttcttcggggcgaaaactctcaaggatcttaccgctgttga
gatccagttcgatgtaacccactcgtgcacccaactgatcttcagcatcttttactttcac-
cagcgtttctgggtgagcaaaaacaggaaggcaaaatgccgcaaaaaagggaataagggcgacacggaaatgttgaatact
catactcttcctttttcaatattattgaagcatttatcagggttattgtctcatgagcg-
gatacatatttgaatgtatttagaaaaataaacaaataggggttccgcgcacatttccccgaaaagtgccacctgacgtctaaga
aaccattattatcatgacattaacctataaaaataggcgtatcac-
gaggccctttcgtctcgcgcgtttcggtgatgacggtgaaaacctctgacacatgcagctcccggagacggtcacagcttgtctg
taagcggatgccgggagcagacaagcccgtcagggcgcgtcagcgggtgtt-
ggcgggtgtcggggctggcttaactatgcggcatcagagcagattgtactgagagtgcaccatatgcggtgtgaaataccgca
cagatgcgtaaggagaaaataccgcatcaggcgccattcgccattcaggctgcgcaactgtt-
gggaagggcgatcggtgcgggcctcttcgctattacgccagctggcgaaagggggatgtgctgcaaggcgattaagttgggt
aacgccagggttttcccagtcacgacgttgtaaaacgacggccagtgccaagctctcgagaa-
gcttactccaagaatatcaaagatacagtctcagaagaccaaagggctattgagacttttcaacaaagggtaatatcgggaaac
ctcctcggattccattgcccagctatctgtcacttcatcaaaaggacag-
tagaaaaggaaggtggcacctacaaatgccatcattgcgataaaggaaaggctatcgttcaagatgcctctgccgacagtggtc
ccaaagatggacccccacccacaaggagcatcgtggaaaaagaagacgttccaaccac-
gtcttcaaagcaagtggattgatgtgatatctccactgacgtaagggatgacgcacaatcccactatccttcgccccaagcttggg
cccaagcttgggtcgcgccccacggatggtataagaataaaggcattccgcgtgcaggat-
tcacccgttcgcctctcaccttttcgctgtactctctcgccacacacacccccctctccagctccgttggagctccggacagcagcagg
cgcggggcggtcacgtagtaagcagctctcggctccctctccccttgctccgtg-
gatccATGGCGCCCACTCTCCCCGTCGTCCCCGCCCACGGGGAGGACAAGAAGAA
GAAGAAGAAGAAGAAGCCGCTCAAGCCTTCGCAGGAGGAGGAG-
GAGGCCCTGCCGCTGCCGCTGCCGCCGCCGGATCGCAAGAGGAAGAAGGCATC
GGAGCCGGTCAACTCGCCGGAGAGAGCCAAGAAGAAGAAGACGGCGAC-
GCCCCAC-
GAGCCGCCATCGGCCAAGCAGCAGAAAAGACCGCTCCCCTTCCAGCGCACGTG
GAGCCCCAACGACGAGGTCCTCATCCTTGAGGCCATGGCGGCGCATCGCCAG-
GAGCACGG-
CAAGGTACCTACCGCCGCGGAGCTCTTCCCCGTCCTCAACGGACGCCTCGACAG
GAAGCGCCTCACCTATAAGAAGCTCGCCGACAAGTTGCGCAC-
CTTCATGCGCCGCCATGG-
CAGAGACGCCAAGAATGGCCCGCCCACCCAAGCGCACGACCGCCGCCTCTACG
ACCTCTCCAGGAATGTCTGGGTGTCTCAAACTCAACCGCCAAACCTAA-
GCGCCAAC-
GCCAATTCAAACATCGCGGGCGGGCAACCCAACCAGCATGATGCCATGCCCAC
CGCAGGCAAGGCGTTCGACAAAATGCGCGACTCCTACCCCAACCTCACCCAA-
GCGCTGTT-
GCTTCTTGTGGGTACGGACCTGGAGAAAGCGCTCACTGCCATTGACGAAACCAA

GGCCCAGGCGCTGGACTTGAAGGTCAGCAACCTCAA-
GAAGGAGCTGTCTGAGGCTGTCATGGAATCGGCAACGATTCAAAGCACAGAAA
GTTCTAAAATACCGTGCTTTCCATCTACCAAGCTTCAACCAGAGTTTGGTG-
CAGAAATTGA-
GAAGAACTTTCAGCTAGAGCATTTGGATGAAATGAAAGGCACCCAAGTGAAATT
GGCAAGAATGGAACAAGAAA-
TATTAGAACTGAAACAAAATTTTTTAGCTTTTCAGTCTCAG-
CAAATGGCTGATAGCAAGCAACAACACGACAAGTCATCAGCGAAGGGTATAAT
ATGTGAATCTTCTGAGAGTGGTCTTCGTTCAATTGTAGCTGACAATAA-
TATTTTATGCAA-
TACTTTGCAAAAGGAGATGGTGGTACAACAGAAATTGTCTTGCGGGAAGACCAA
GGAGGTTACTTCTAAGCATCGACATCCTCAGAAGCTGGTTGTCTTTCCTTTT-
ggatccatggtgagcaagggcgaggagctgttcaccggggtggtgcccatcctggtcgagctggacggcgacgtaaacggc
cacaagttcagcgtgtccggcgagggcgagggcgatgccacctacggcaa-
gctgaccctgaagttcatctgcaccaccggcaagctgcccgtgccctggcccaccctcgtgaccaccttcacctacggcgtgcag
tgcttcagccgctaccccgaccacatgaagcagcac-
gacttcttcaagtccgccatgcccgaaggctacgtccaggagcgcaccatcttcttcaaggacgacggcaactacaagacccgc
gccgaggtgaagttcgagggcgacaccctggtgaaccgcatcgagctgaaggg-
catcgacttcaaggaggacggcaacatcctggggcacaagctggagtacaactacaacagccacaacgtctatatcatggccg
acaagcagaagaacggcatcaaggtgaacttcaagatccgccacaacatcgaggacgg-
cagcgtgcagctcgccgaccactaccagcagaacacccccatcggcgacggccccgtgctgctgcccgacaaccactacctga
gcacccagtccgccctgagcaaagaccccaacgagaagcgcgatcacatggtcctgctggag-
ttcgtgaccgccgccgggatcactcacggcatggacgagctgtacaagtaaagcggccgcccggctgcagatcgttcaaacatt
tggcaataaagtttcttaagattgaatcctgttgccggtcttgcgatgattatcat-
atatatttctgttgattacgttaagcatgtaataattaacatgtaatgcatgacgttatttatgagatgggtttttatgattagagtccc
gcaattatacatttaatacgcgatagaaaacaaaatatagcgcgcaaactagga-
taaattatcgcgcgcggtgtcatctatgttactagatc

The construct map of transient expression vector 35s:OsGeBP11-eGFP

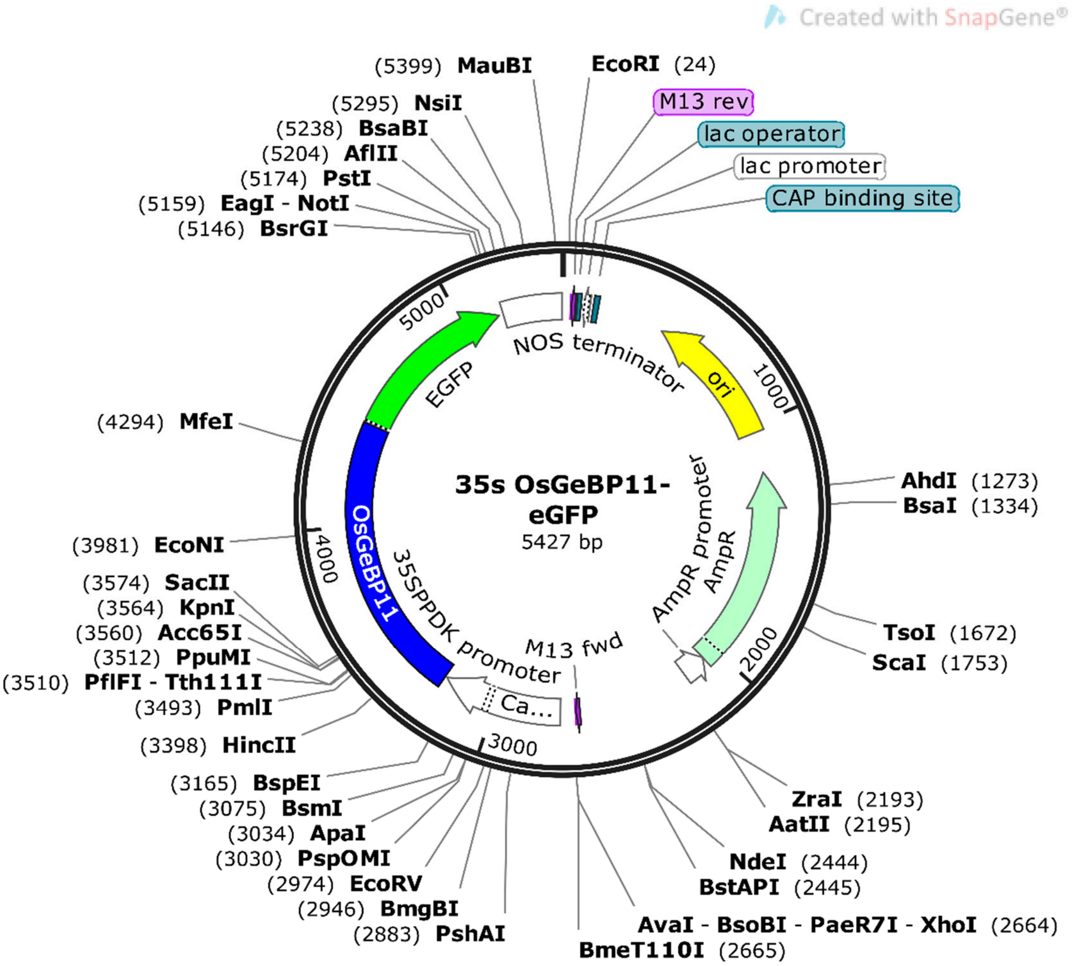

Figure S11. The construct map of transient expression vector 35s:OsGeBP11-eGFP. The 35s:OsGeBP11-eGFP vector was constructed using the PUC18 plasmid as a backbone. The full length of O. sativa ssp. Japonica OsGeBP11 cDNA was used to generate this construct.

>nucleotide construct for transient expression vector of 35s:OsGeBP12-eGFP
cgatgataagctgtcaaacatgagaattcg-
taatcatggtcatagctgtttcctgtgtgaaattgttatccgctcacaattccacacaacatacgagccggaagcataaagtgtaaa
gcctggggtgcctaatgagtgagctaactcacattaattgcgttgcgctcac-
tgcccgctttccagtcgggaaacctgtcgtgccagctgcattaatgaatcggccaacgcgcggggagaggcggtttgcgtattg
ggcgctcttccgcttcctcgctcac-
tgactcgctgcgctcggtcgttcggctgcggcgagcggtatcagctcactcaaaggcggtaatacggttatccacagaatcagg
ggataacgcaggaaagaacatgtgagcaaaaggccagcaaaaggccaggaaccg-
taaaaaggccgcgttgctggcgtttttccataggctccgcccccctgacgagcatcacaaaaatcgacgctcaagtcagaggtg
gcgaaacccgacaggactataaagataccaggcgtttccccctggaa-
gctccctcgtgcgctctcctgttccgaccctgccgcttaccggatacctgtccgcctttctcccttcgggaagcgtggcgctttctcat
agctcacgctgtaggtatctcagttcggtgtaggtcgttcgctccaagctgggctgtgtg-
cacgaaccccccgttcagcccgaccgctgcgccttatccggtaactatcgtcttgagtccaacccggtaagacacgacttatcgcc
actggcagcagccactggtaacaggattagcagagcgaggtatgtaggcggtgctacagag-
ttcttgaagtggtggcctaactacggctacactagaaggacagtatttggtatctgcgctctgctgaagccagttaccttcggaaa
aagagttggtagctcttgatccggcaaacaaaccaccgctggtagcggtggtttttttgttt-
gcaagcagcagattacgcgcagaaaaaaaggatctcaagaagatcctttgatcttttctacggggtctgacgctcagtggaacg
aaaactcacgttaagggattttggtcatgagattatcaaaaaggatcttcaccta-
gatccttttaaattaaaaatgaagttttaaatcaatctaaagtatatatgagtaaacttggtctgacagttaccaatgcttaatcagtg
aggcacctatctcagcgatctgtctatttcgttcatccatagttgcctgactccccgtcgtg-
tagataactacgatacgggagggcttaccatctggccccagtgctgcaatgataccgcgagacccacgctcaccggctccagat

ttatcagcaataaaccagccagccggaagggccgagcgcagaagtggtcctg-
caactttatccgcctccatccagtctattaattgttgccgggaagctagagtaagtagttcgccagttaatagtttgcgcaacgttgt
tgccattgctacaggcatcgtggtgtcacgctcgtcgtttgg-
tatggcttcattcagctccggttcccaacgatcaaggcgagttacatgatcccccatgttgtgcaaaaaagcggttagctccttcgg
tcctccgatcgttgtcagaagtaagttggccgcagtgttatcactcatggttatggcagcac-
tgcataattctcttactgtcatgccatccgtaagatgcttttctgtgactggtgagtactcaaccaagtcattctgagaatagtgtatg
cggcgaccgagttgctcttgcccggcgtcaatacgggataatac-
cgcgccacatagcagaactttaaaagtgctcatcattggaaaacgttcttcggggcgaaaactctcaaggatcttaccgctgttga
gatccagttcgatgtaacccactcgtgcacccaactgatcttcagcatcttttactttcac-
cagcgtttctgggtgagcaaaaacaggaaggcaaaatgccgcaaaaaagggaataagggcgacacggaaatgttgaatact
catactcttcctttttcaatattattgaagcatttatcagggttattgtctcatgagcg-
gatacatatttgaatgtatttagaaaaataaacaaataggggttccgcgcacatttccccgaaaagtgccacctgacgtctaaga
aaccattattatcatgacattaacctataaaaataggcgtatcac-
gaggccctttcgtctcgcgcgtttcggtgatgacggtgaaaacctctgacacatgcagctcccggagacggtcacagcttgtctg
taagcggatgccgggagcagacaagcccgtcagggcgcgtcagcgggtgtt-
ggcgggtgtcggggctggcttaactatgcggcatcagagcagattgtactgagagtgcaccatatgcggtgtgaaataccgca
cagatgcgtaaggagaaaataccgcatcaggcgccattcgccattcaggctgcgcaactgtt-
gggaagggcgatcggtgcgggcctcttcgctattacgccagctggcgaaagggggatgtgctgcaaggcgattaagttgggt
aacgccagggttttcccagtcacgacgttgtaaaacgacggccagtgccaagctctcgagaa-
gcttactccaagaatatcaaagatacagtctcagaagaccaaagggctattgagacttttcaacaaagggtaatatcgggaaac
ctcctcggattccattgcccagctatctgtcacttcatcaaaaggacag-
tagaaaaggaaggtggcacctacaaatgccatcattgcgataaaggaaaggctatcgttcaagatgcctctgccgacagtggtc
ccaaagatggacccccacccacaaggagcatcgtggaaaaagaagacgttccaaccac-
gtcttcaaagcaagtggattgatgtgatatctccactgacgtaagggatgacgcacaatcccactatccttcgccccaagcttggg
cccaagcttgggtcgcgccccacggatggtataagaataaaggcattccgcgtgcaggat-
tcacccgttcgcctctcaccttttcgctgtactctctcgccacacacaccccctctccagctccgttggagctccggacagcagcagg
cgcggggcggtcacgtagtaagcagctctcggctccctctccccttgctccgtg-
gatccATGAGCTCATGGTGGTCGTGGCGGAGCCTCTTCTCCTCCTTGGCCAACGCCA
ACGGAGGGGGCAGCAATGCCGATGCTTCCTCCGGATCCGGCAC-
CTCCTCCTCCCCTCCCGTCCATGAAGCTCAACAAGCAGCAGCTCGCCGGCGAAG
CGCTCGCACCAAGAAGCCTCCCGAGGAAGAAGCAGCAGGATCGCAGCCG-
CAGCCCAA-
GACTCGTCCTTCACCGGCGTCCAAGGCGTCCAAGGCGAAGGTGCTGCTGCTGCT
CGGCGACGGCGAGCCCAAGAAGAA-
GCCAGCCCCGAACCCGACCCCGACTCAGAAGCGTAG-
CAACAAGAGAAAGCGGTCGTGGTCCAGGGCCGACGAGCTCAGGATTCTGGAGG
CCATGGCCAACCACGCCAACGCCCATGGCGGCGCTCTGCCG-
GAGGCCTCCGATCTCTTT-
GCCGCCCTTGCCAGCAGCCTCGAGAGAGGAGATGCCGACCTGCCCAAGCTCGCC
GACAAGGTCCACAAGCTCAAGAGATGGTATGACAATGCACGCCTGCCG-
CAGCGCTGCCCGACTGACGATGACGACGATACGCGTCGATTGTTCCAGCTATGT
GGGAAGGTTTGGGGTCCCCCTTCAACTGTGCTGCGCACGAGCCCACGCCAAC-
GCCACAAGGTAGTAGGAGTACTTGTCCAAGGAAATGGTGCCAATCCCCAGCCG
GCAGCAGCACTCAAGGTCAAGGAGAAGAGAGTCAGGAGGGAG-
TTATCTGAGCTTTATGTCCTGTATCCTTGTCTCGCCCAGGAGGTCAAGGCGCATG
CTAACGAATACGGCGAGCTCATCGGAACAGCCTTTCAGTTCATCGGCGATGAC-
GAA-
GCTCGATGTTACGACGACAGGTACAGGAAGATGTTGGTGGACAAGCTTAACATG
AAGAAGGAACATGCCGACGTAACAAAGTCCCTGCTGTGCACTCTTGCAGGC-
TACATAAATggatccatggtgagcaagggcgaggagctgttcaccggggtggtgcccatcctggtcgagctggacgg
cgacgtaaacggccacaagttcagcgtgtccggcgagggcgagggcgatgccacctacgg-
caagctgaccctgaagttcatctgcaccaccggcaagctgcccgtgccctggcccaccctcgtgaccaccttcacctacggcgtg
cagtgcttcagccgctaccccgaccacatgaagcagcac-
gacttcttcaagtccgccatgcccgaaggctacgtccaggagcgcaccatcttcttcaaggacgacggcaactacaagacccgc

gccgaggtgaagttcgagggcgacaccctggtgaaccgcatcgagctgaaggg-
catcgacttcaaggaggacggcaacatcctggggcacaagctggagtacaactacaacagccacaacgtctatatcatggccg
acaagcagaagaacggcatcaaggtgaacttcaagatccgccacaacatcgaggacgg-
cagcgtgcagctcgccgaccactaccagcagaacacccccatcggcgacggccccgtgctgctgcccgacaaccactacctga
gcacccagtccgccctgagcaaagaccccaacgagaagcgcgatcacatggtcctgctggag-
ttcgtgaccgccgccgggatcactcacggcatggacgagctgtacaagtaaagcggccgcccggctgcagatcgttcaaacatt
tggcaataaagtttcttaagattgaatcctgttgccggtcttgcgatgattatcat-
atatatttctgttgattacgttaagcatgtaataattaacatgtaatgcatgacgttatttatgagatgggtttttatgattagagtccc
gcaattatacatttaatacgcgatagaaaacaaaatatagcgcgcaaactagga-
taaattatcgcgcgcggtgtcatctatgttactagatc

The construct map of transient expression vector 35s:OsGeBP12-eGFP

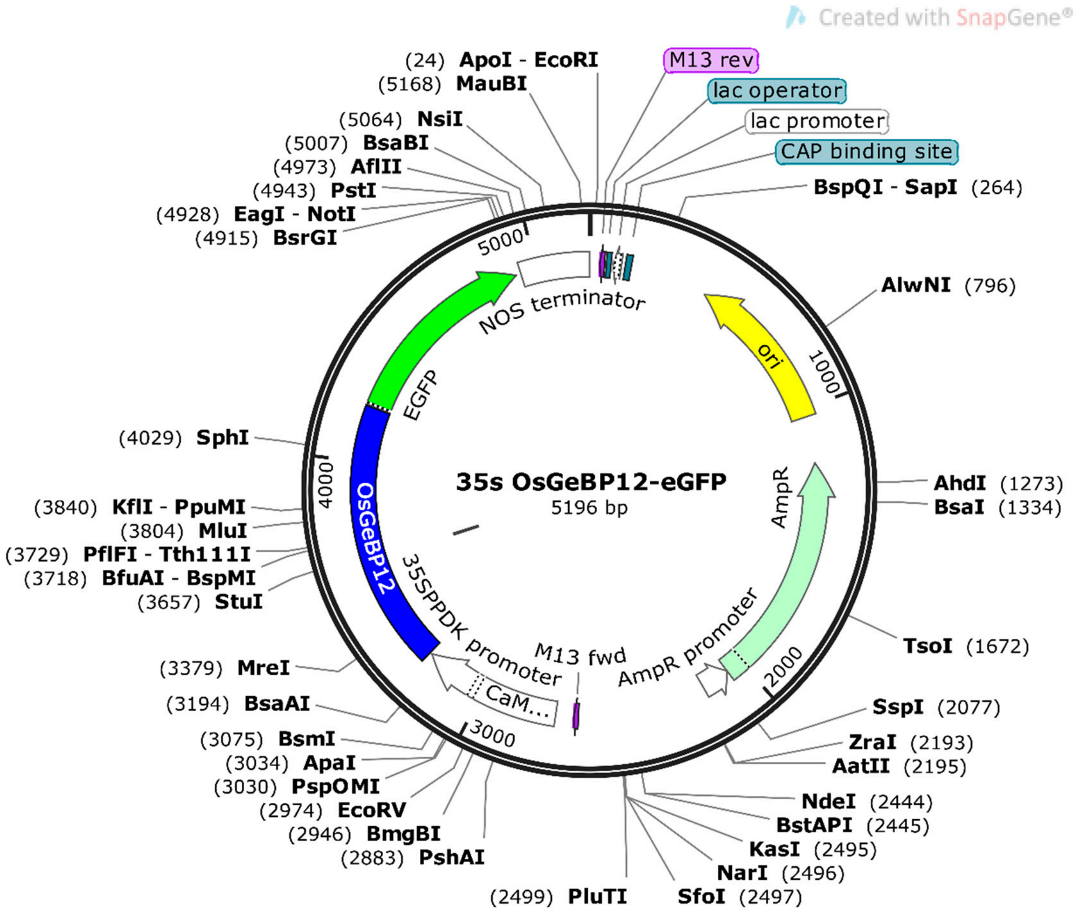

Figure S12. The construct map of transient expression vector 35s:OsGeBP12-eGFP. The 35s:OsGeBP12-eGFP vector was constructed using the PUC18 plasmid as a backbone. The full length of O. sativa ssp. Japonica OsGeBP12 cDNA was used to generate this construct.
